# Pan-species genome-wide evolution of the RGF peptide gene family and its impact on adventitious root regeneration in apple

**DOI:** 10.1093/hr/uhag151

**Published:** 2026-04-28

**Authors:** Yue Wang, Yupeng Zhang, Yu Zhang, Hao Tan, Lin Liu, Shuxin Xu, Jinghua Qu, Yafei Shu, Xiaoliang Wu, Ruirui Xu, Shizhong Zhang

**Affiliations:** College of Life Sciences, Shandong Agricultural University, Taiʼan 271018, China; College of Life Sciences, Shandong Agricultural University, Taiʼan 271018, China; College of Life Sciences, Shandong Agricultural University, Taiʼan 271018, China; College of Life Sciences, Shandong Agricultural University, Taiʼan 271018, China; College of Life Sciences, Shandong Agricultural University, Taiʼan 271018, China; College of Life Sciences, Shandong Agricultural University, Taiʼan 271018, China; College of Life Sciences, Shandong Agricultural University, Taiʼan 271018, China; College of Life Sciences, Shandong Agricultural University, Taiʼan 271018, China; College of Biology and Brewing Engineering, Taishan University, Tai’an, Shandong 271000, China; College of Biology and Oceanography, Weifang University, Weifang, Shandong 261061, China; College of Life Sciences, Shandong Agricultural University, Taiʼan 271018, China

## Abstract

Root meristem growth factors (RGFs) are pivotal regulators of root growth in plants and are critically involved in the regulation of root development in fruit trees. In this study, we constructed a comprehensive small secreted peptide library from 64 plant species. Using a structure-based screening strategy, we identified a total of 495 *RGF* genes. Phylogenetic analysis revealed that the modern *RGF* gene family originated in ferns and exhibited an evolutionary pattern of initial contraction and subsequent expansion, consistent with the evolutionary emergence and diversification of plant root systems. Analysis of the *Malus* pan-genome indicated that *RGF* genes within the genus were classified into 12 clades, with some clades present exclusively in wild *Malus* species. Notably, the repertoire and copy number of RGF peptides vary significantly among *Malus* species with different ploidy levels, suggesting a potential association between *RGF* gene expansion and polyploidization. Functional analysis of a gene encoding the canonical RGF mature peptide ‘DYTPARKKPPIHN’ in *Malus* demonstrated a dose-dependent effect on primary root elongation in apple seedlings following exogenous application. Subsequent experiments confirmed that overexpression of *MdglRGF1* negatively regulates adventitious root growth during regeneration-associated and hormone-induced adventitious rooting, a phenotype sustained throughout adventitious root development in soil-grown seedlings. Collectively, our research provides novel insights into the identification and functional characterization of small peptides and establishes a foundation for their potential application in apple rootstock breeding and the development of ideal root system architecture.

## Introduction

Secreted small peptides function as critical hormonal signals governing intercellular communication and coordinating a wide array of plant growth and developmental processes [1]. Based on their structural features, these secreted peptide hormones are generally classified into two major groups: small post-translationally modified peptides (SMPs) and cysteine-rich peptides (CRPs) [2]. Gene families encoding post-translationally modified peptides often arise and expand through genomic events such as gene duplication and polyploidization [3]. Reflecting their functional importance, the *Arabidopsis* genome alone harbors numerous small peptide gene families predicted to encode over one thousand secreted peptides [4], which play important regulatory roles in various aspects of plant biology [5, 6].

Root meristem growth factors (RGFs), also known as GOLVEN (GLV) or CLE-like (CLEL) peptides, constitute a conserved family of tyrosine-sulfated, post-translationally modified peptide hormones (hereafter RGF peptides). Initially identified independently in *Arabidopsis thaliana* [7, 8], a functional RGF peptide typically consists of a 13-amino-acid fragment derived from C-terminal processing of a precursor protein exceeding 100 amino acids [9, 10]. The *RGF* gene family has since been characterized in several plant species, including *Arabidopsis* [9], ginseng (*Panax ginseng*) [11], and Japanese knotweed (*Fallopia japonica*) [12]. Notably, a single, highly conserved RGF-like peptide identified in bryophytes exhibits growth-regulatory functions analogous to those of vascular plant RGFs, indicating an ancient evolutionary origin [13].

Extensive functional studies in *Arabidopsis* have established that RGF peptides are essential for maintaining root meristem activity, as evidenced by the reduced root apical meristem size in *rgf1,2,3* multiple mutants. In ginseng, PgRGF1 influences both primary and adventitious root development [11]. The ability of peptide hormones to shape root system architecture, first demonstrated in *Arabidopsis* and subsequently observed across diverse species [14–16], underscores their fundamental role. In apple (*Malus domestica*), a globally important fruit crop propagated predominantly through grafting [17, 18], elucidating the functions of small peptide hormones like RGFs in root development is of direct agronomic relevance for rootstock improvement and productivity [19]. The recent release of the *Malus* pan-genome now provides an unprecedented opportunity to investigate the evolutionary dynamics and natural variation of the *RGF* gene family across apple species on a genomic scale [20].

In this study, we performed a systematic, phylogeny-informed identification of *RGF* genes across 64 plant species via a dedicated secreted peptide library and motif-based screening, leading to the discovery of 495 family members. Our evolutionary analysis delineated the origin and diversification pattern of the family. Leveraging the *Malus* pan-genome, we revealed the phylogenomic structure, polyploidy-associated expansion, and domestication-driven contraction of the *RGF* family within apple. Through functional characterization, we demonstrate that exogenous application of a synthetic RGF peptide to seedlings of the apomictic rootstock *Malus hupehensis* exerts a biphasic dose-dependent effect on primary root growth and strongly suppresses lateral root formation. This dose–response mechanism was conserved in *A. thaliana*. Furthermore, genetic manipulation of *MdglRGF1* expression in the model cultivar ‘Gala’ not only modulates adventitious rooting across developmental contexts but also impacts shoot morphology, indicating systemic regulatory roles. Our work establishes a robust framework for small peptide discovery and functional analysis, providing both mechanistic insights and a genetic resource for leveraging peptide signaling in apple rootstock breeding and the design of optimized root systems.

## Results

### Construction of a pan-species genomic secreted peptide library

**Figure 1:**
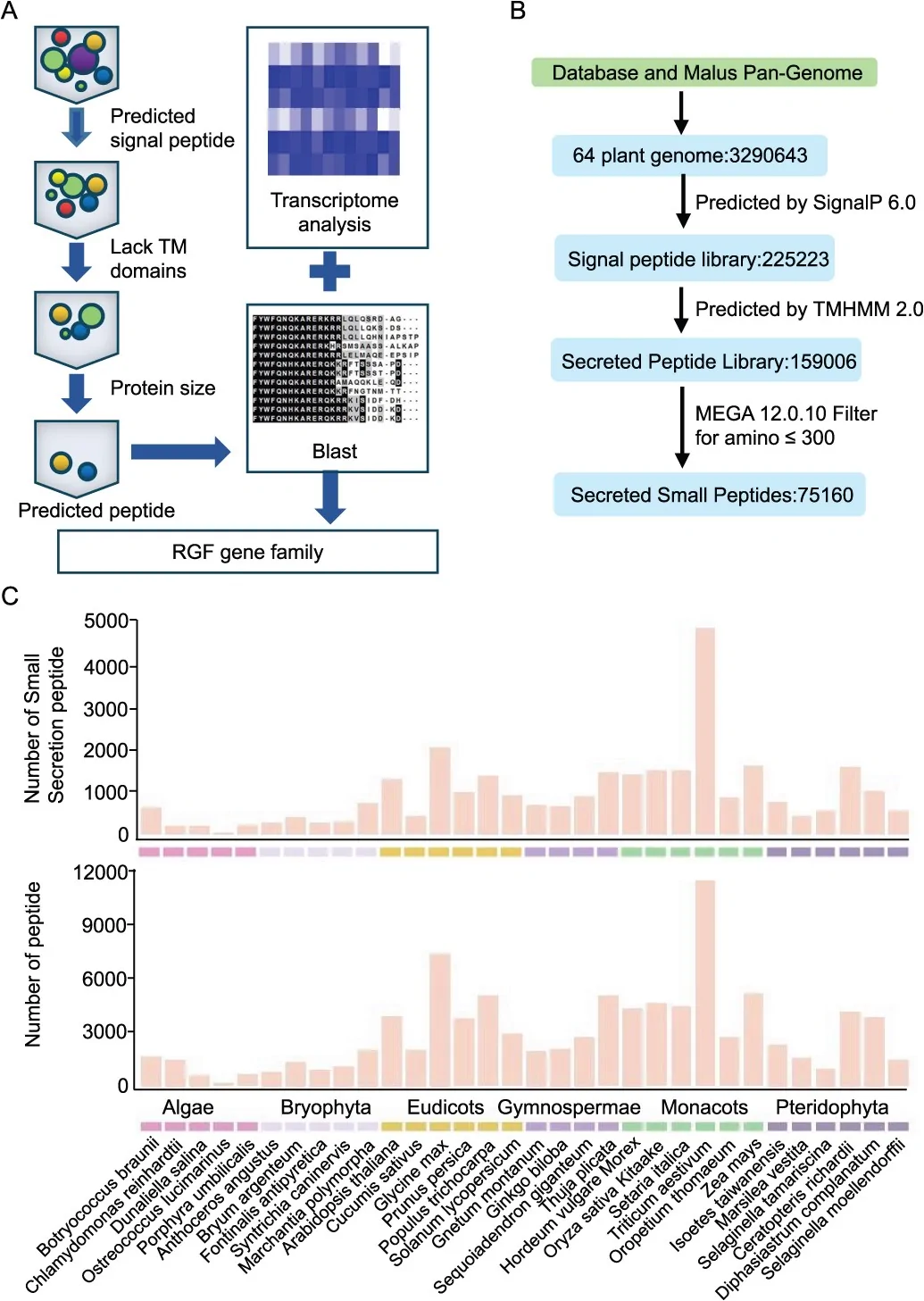
Construction of a multi-species small secreted peptide library at the pan-genome level. (A) Construction process of the small secretory peptide library. (B) Variations in software and peptide quantities during secretory peptidome library construction. (C) Statistics of the number of proteins and small secretory peptides in various species at the pangenome level.

To comprehensively identify the diversity and abundance of RGF peptides in plants, we employed a bioinformatic strategy of ‘global library construction followed by in-library annotation’ for multi-species small secreted peptide discovery. First, we constructed a multi-species signal peptide library based on the defining features of secreted peptides. We utilized genomic data from publicly available databases and the *Malus* pan-genome, encompassing 5 algal species, 5 bryophytes, 6 ferns, 4 gymnosperms, 6 monocots, 6 dicots, and 32 *Malus* species (64 species in total) to represent the phylogenetic diversity of land plants and the population characteristics within the genus *Malus* (Table S1), which were used to predict and filter for N-terminal signal peptides (which direct peptides into the secretory pathway), thereby constructing a signal peptide library for the studied plants (Table S2). Peptides with a predicted signal peptide and no transmembrane domains were defined as secreted peptides and compiled into a secreted peptide library (Table S3). Given that previously reported RGF peptide precursors typically range from 39 to 277 amino acids in length, we filtered the secreted peptides to generate a candidate small secreted peptide library enriched for potential RGF peptides (Fig. 1A; Table S4). To infer the potential functions of these candidate peptides, we performed Gene Ontology (GO) enrichment analysis on the predicted small secreted peptides from the apple cultivar ‘Gala’. GO enrichment analysis revealed significant enrichment of terms related to ‘plant-type cell wall organization or biogenesis’ and ‘growth factor activity’, suggesting roles in apple immune responses and growth regulation (Fig. S1A; Table S5). Our small secreted peptide library provides a foundational resource for the subsequent screening of peptides with defined functional motifs (Fig. 1B). We next visualized the relationship between the number of small secreted peptides and the total peptide count per species revealed a general positive correlation across most plants (Fig. 1C). However, the triploid apple species *Malus × platycarpa* exhibited a notable deviation in the proportion of signal peptides (Fig. S1B). Given the established key role of RGF peptides in root development [21, 22], we focused on peptides specifically expressed in apple roots. By integrating root transcriptome data from the apple cultivar ‘Gala’, we annotated the expression patterns of the secreted peptides. We found that the expression levels of small secreted peptides exhibited dynamic variation among individuals at the same developmental stage (Fig. S2A). Subsequently, we specifically screened small peptides stably expressed in the roots of ‘Gala’ apple (detected in all three biological replicates) and performed GO enrichment analysis on these peptides (Table S6). A similar expression pattern was observed for root-stably expressed peptides (Fig. S2B). GO enrichment analysis of these apple root-specific small secreted peptides revealed significant terms related to the ‘extracellular region’, ‘immune response’, ‘defense response’, and ‘metabolic processes’ (Fig. S3A), consistent with their involvement in plant growth and stress-related processes.

### Phylogenetic origin and evolutionary diversification of the RGF gene family in land plants

The primary structure of RGF precursor peptides typically comprises three domains: an N-terminal hydrophobic signal peptide, a conserved motif at the C terminus representing the mature RGF peptide, and a variable nonconserved region connecting the two [7, 23, 24]. Based on this structural characteristic and the previously constructed secreted peptide library, we employed Hidden Markov ModelER (HMMER) and Basic Local Alignment Search Tool (BLAST)-based methods to identify candidate RGF peptides [25], across a broad range of plant species and within the genus *Malus* (Tables S7 and S8). We performed a systematic phylogenetic analysis of the *RGF* gene family across 32 representative land plant species. Given the pivotal role of ferns in the evolution of root organs, we expanded the selection of fern genomes for this analysis. The selected species included five algae (*Botryococcus braunii*, *Chlamydomonas reinhardtii*, *Dunaliella salina*, *Ostreococcus lucimarinus*, *Porphyra umbilicalis*), five bryophytes (*Anthoceros angustus*, *Marchantia polymorpha*, *Fontinalis antipyretica*, *Bryum argenteum*, *Syntrichia caninervis*), six ferns (*Selaginella tamariscina*, *S. moellendorffii*, *Diphasiastrum complanatum*, *Isoetes taiwanensis*, *Marsilea vestita*, *Ceratopteris richardii*), four gymnosperms (*Ginkgo biloba*, *Sequoiadendron giganteum*, *Gnetum montanum*, *Thuja plicata*), six eudicots (*A. thaliana*, *Cucumis sativus*, *Glycine max*, *Prunus persica*, *Populus trichocarpa*, *Solanum lycopersicum*), and six monocots (*Hordeum vulgare* ‘Morex’, *Oryza sativa* ‘Kitaake’, *Setaria italica*, *Triticum aestivum*, *Zea mays*, *Oropetium thomaeum*) to represent major evolutionary lineages of green plants. Through a combined approach of library screening, BLAST homology searches against known *Arabidopsis RGF* genes, and manual curation of mature peptide sequences, we identified 161 RGF members distributed across 22 of these species (Table 1). Notably, we observed sequences containing RGF-like conserved motifs in *A. angustus*, *B. argenteum*, and *M. polymorpha*. However, as these were not predicted to be secreted peptides within our library, they were not included in the final count of *RGF* gene family members—consistent with previous reports [13].

**Table 1:** Gene numbers of three clades of RGF Peptide gene family of all species.

**Category**	**Species**	**Number of DY conservative motifs**	**RGF family number**	**Ancient clade**	**Core clade**	**Luxury clade**
Algae	*Botryococcus braunii*	0	0	0	0	0
	*Chlamydomonas reinhardtii*	0	0	0	0	0
	*Dunaliella salina*	0	0	0	0	0
	*Ostreococcus lucimarinus*	0	0	0	0	0
	*Porphyra umbilicalis*	0	0	0	0	0
Bryophyta	*Anthoceros angustus*	1	0	0	0	0
	*Marchantia polymorpha*	1	0	0	0	0
	*Fontinalis antipyretica*	0	0	0	0	0
	*Bryum argenteum*	1	0	0	0	0
	*Syntrichia caninervis*	0	0	0	0	0
Pteridophyta	*Diphasiastrum complanatum*	3	7	7	0	0
	*Selaginella moellendorffii*	5	6	4	2	0
	*Isoetes taiwanensis*	2	2	1	1	0
	*Marsilea vestita*	1	1	1	0	0
	*Ceratopteris richardii*	12	19	19	0	0
Gymnospermae	*Thuja plicata*	2	4	4	0	0
	*Gnetum montanum*	2	4	2	0	1
	*Ginkgo biloba*	2	2	2	0	0
	*Sequoiadendron giganteum*	3	3	3	0	0
Monocots	*Hordeum vulgare Morex*	6	7	3	2	2
	*Oryza sativa Kitaake*	7	8	4	3	1
	*Setaria italica*	6	8	4	2	2
	*Triticum aestivum*	4	11	2	7	2
	*Zea mays*	5	8	2	5	1
	*Oropetium thomaeum*	4	4	2	2	0
Eudicots	*Arabidopsis thaliana*	10	10	5	5	0
	*Cucumis sativus*	6	6	2	4	0
	*Glycine max*	13	19	2	13	4
	*Prunus persica*	7	8	0	6	2
	*Populus trichocarpa*	10	17	4	9	4
	*Solanum lycopersicum*	7	7	0	4	3

**Figure 2:**
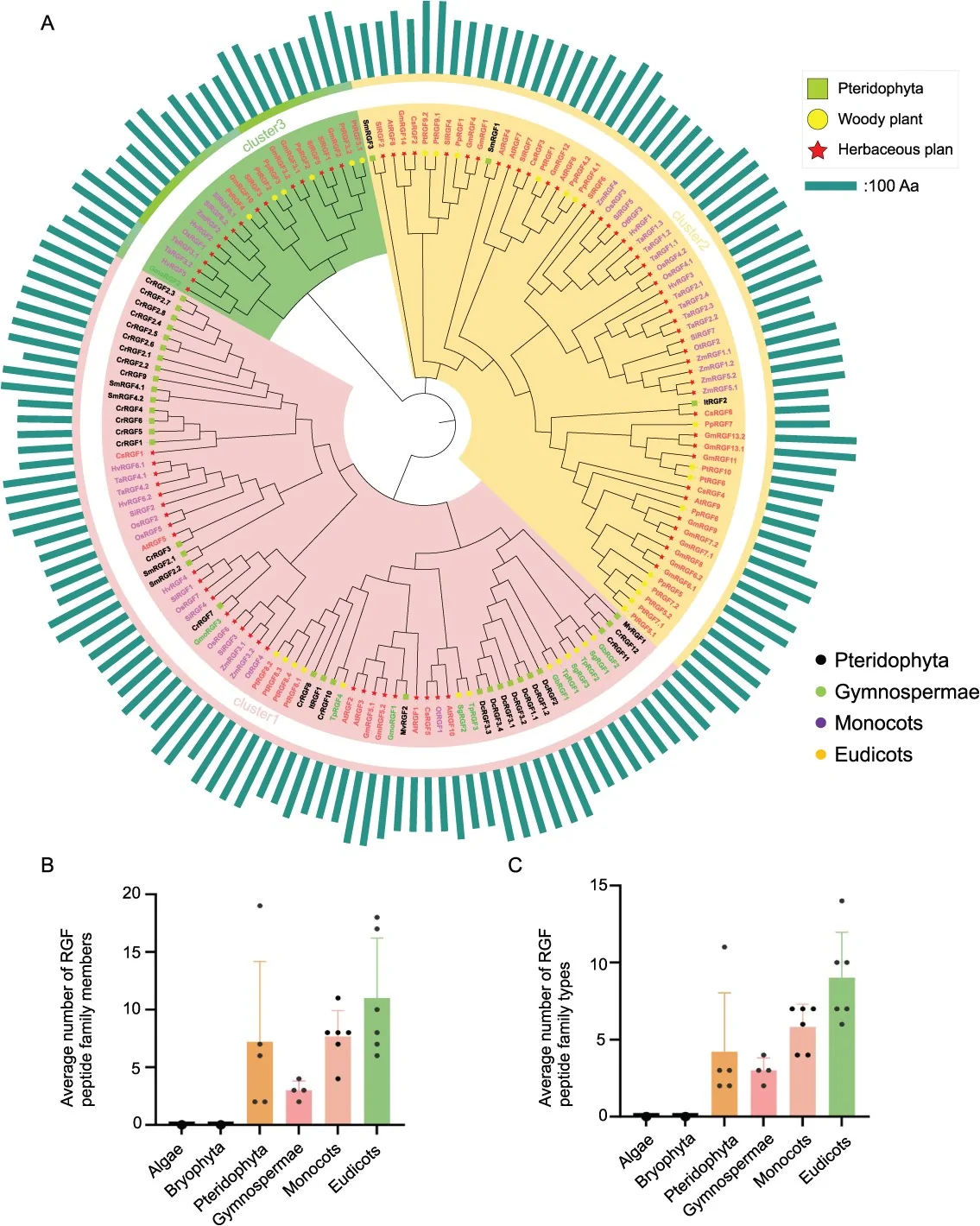
Evolution of the RGF peptide family in natural species. (A) Phylogenetic relationships of the *RGF* gene family. The phylogenetic tree is divided into three distinct clusters of the RGF gene family: the ancient clade (cluster 1), the Core clade (cluster 2), and the Luxury clade (cluster 3). The inner-ring labels of the phylogenetic tree indicate different plant species, where ‘cr’ denotes *Ceratopteris richardii*, ‘It’ denotes *Isoetes taiwanensis*, ‘Mv’ denotes *Marsilea vestita*, ‘Sm’ denotes *Selaginella moellendorffii*, ‘DC’ denotes *Diphasiastrum complanatum*, ‘Tp’ denotes *Thuja plicata,* ‘Gb’ denotes *Ginkgo biloba*, ‘Gmo’ denotes *Gnetum montanum*, ‘Sg’ denotes *Sequoiadendron giganteum*, ‘Ot’ denotes *Oropetium thomaeum*, ‘Si’ denotes *Setaria italica*, ‘Ta’ denotes *Triticum aestivum*, ‘Zm’ denotes *Zea mays*, ‘Hv’ denotes *Hordeum vulgare Morex*, ‘Os’ denotes *Oryza sativa Kitaake*, ‘Sl’ denotes *Solanum lycopersicum*, ‘Pp’ denotes *Prunus persica*, ‘Pt’ denotes *Populus trichocarpa*, ‘At’ denotes *Arabidopsis thaliana*, ‘Cs’ denotes *Cucumis sativus*, and ‘Gm’ denotes *Glycine max*. The outermost bar graph represents the original peptide length of RGFs in different plant species. (B) Average number of RGF peptide family members in Natural species. (C) Average number of RGF peptide family types.

We constructed a phylogenetic tree using the RGF sequences identified from these species (Fig. 2A). Evolutionary analysis revealed that the *RGF* gene family is divided into three distinct clades during the course of evolution. We defined Cluster 1, which is predominantly composed of fern-derived homologs, as the Ancient clade. Cluster 2, which contains the majority of *RGF* family members in modern plants, was defined as the Core clade. Cluster 3, which exhibits low homology to fern RGFs, was consequently defined as the Luxury clade. Notably, within the Ancient clade, the *RGF* family of the lycophyte *D. complanatum* is separated from other ferns. This pattern may reflect the relatively slow evolutionary rate of lycophytes, as indicated in prior studies [26]. Within the Core clade, monocots and eudicots form clearly distinct evolutionary clusters, indicating that the *RGF* gene family underwent co-evolution alongside the divergence of monocots and eudicots. Furthermore, the *RGF* family in modern plants likely evolved from homologs present in *S. moellendorffii* and *I. taiwanensis*. We quantified the amino acid length of these RGF precursor peptides. Their average length showed little variation across different plant phyla [27], consistently ranging between 100 and 150 amino acids (Fig. S4A). This observation informed subsequent precursor peptide screening criteria. Consistent with previous studies, we observed RGF-like motifs in bryophytes. However, the *RGF* family possessing a complete signal peptide structure originated in ferns (Fig. 2B). By using different RGF conserved motifs to represent distinct RGF subfamilies, we found that during plant evolution, RGF subfamilies exhibited a pattern of rapid emergence and contraction, followed by a new round of expansion observed in modern eudicots (Fig. 2C). This aligns with previous research indicating that polyploidization events in eudicots led to the expansion of gene families [28].

### Pan-genome level systematic identification and analysis of the *RGF* gene family in *Malus*

Cultivated apple (*M. domestica*), a fruit tree crop with a long history of domestication, has undergone directional selection for superior fruit traits such as larger size and lower acidity, with resource allocation shifting toward above-ground reproductive growth [29]. In contrast, wild apples, which must adapt to complex natural environments, tend to allocate a greater proportion of root system development. Additionally, the prevalence of polyploidy within the genus *Malus* is associated with enhanced adaptive capacity to diverse environments [30]. These factors may have contributed to the diversification of the *RGF* family. Using the published *Malus* pan-transcriptome, we conducted a genome-wide analysis of the *RGF* gene family across 32 *Malus* accessions. This set included 19 wild diploids (*Eriolobus florentinus*, *Malus baccata*, *M. doumeri*, *M. floribunda*, *M. fusca*, *M. halliana*, *M. honanensis*, *M. ioensis*, *M. komarovii*, *M. mandshurica*, *M. melliana*, *M. micromalus*, *M. ombrophila*, *M. orientalis*, *M. prunifolia*, *M. sargentii*, *M. spectabilis*, *M. yunnanensis*, *M. zumi*), 5 wild tetraploids (*M. asiatica*, *M. baccata* var. *xiaojinensis*, *M. rockii*, *M. sieboldii*, *M. sikkimensis*), 4 wild triploids (*M. angustifolia*, *M. hupehensis*, *M. transitoria*, *M. × platycarpa*), and 4 cultivated diploids (*M. domestica* ‘Fuji’, *M. domestica* ‘Gala’, *M. domestica* ‘GDDH,’ *M. domestica* ‘Hanfu’). We identified 334 *RGF* family members within the genus. *RGF* genes were renamed sequentially according to their chromosomal positions. Phylogenetic analysis indicated that these *RGF* genes were classified into 12 clades based on their encoded mature RGF peptides (Fig. S5).

**Figure 3:**
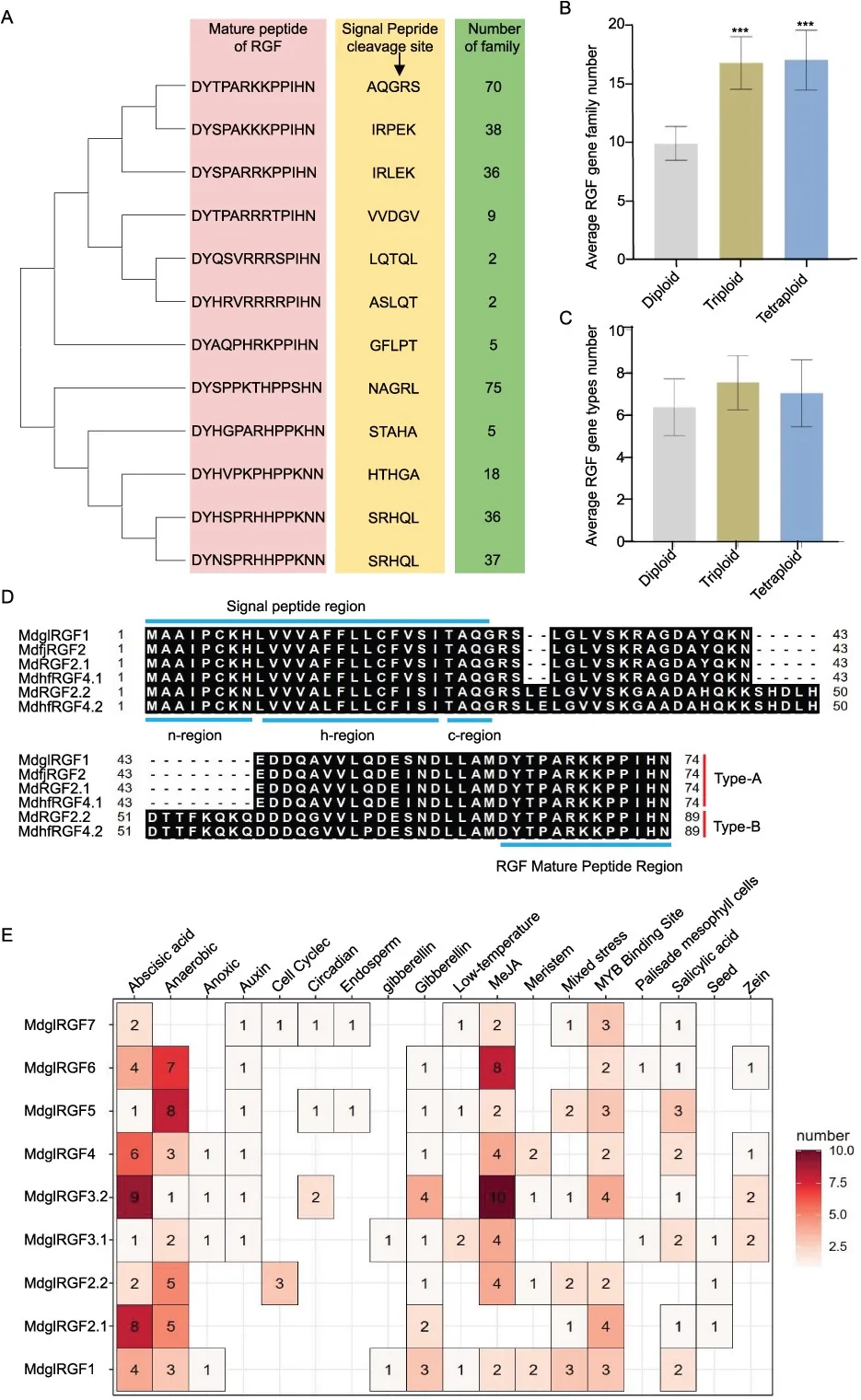
The phylogenetic clustering, gene copy number, and clade diversity variations, precursor protein sequence conservation, and promoter *cis*-regulatory element profiles of the *RGF* gene family across Malus accessions. (A) Phylogenetic tree of *Malus RGF* genes (clustered by mature peptide sequences), with clade-specific mature peptides, signal peptide regions, and gene counts annotated. (B, C) The number of *RGF* gene family and types in different *Malus* groups. (D) Multiple sequence alignment of *RGF* precursors (encoding ‘DYTPARKKPPIHN’), showing conserved regions (signal peptide, variable region, mature peptide) and Type A/B classification. (E) Heatmap of *cis*-regulatory elements in *RGF* promoters across *Malus* species, with color intensity indicating element counts per promoter.

The two largest clades encode mature peptides with the sequences ‘DYTPARKKPPIHN’ and ‘DYSPPKTHPPSHN’, comprising 70 and 75 members, respectively (Fig. 3A). In other land plant species, these two conserved motifs have only been found in peach, tomato, and poplar, residing within the Core clade and the Luxury clade, respectively. The *RGF* gene family encoding the mature peptide ‘DYAQPHRKPPIHN’ was detected exclusively in the four cultivated varieties and one wild species, *M. ioensis*. Sequence alignment revealed high protein sequence conservation within this clade (Fig. S6). We further compared the average number of RGF clades (diversity) and the total copy number of *RGF* genes among different *Malus* groups (Fig. S7A and B). Compared to wild apples, cultivated apples exhibited a contraction in the *RGF* gene family. Among *Malus* accessions of different ploidy levels, while the average clade diversity shows little variation, the total gene copy number differs significantly (Fig. 3B and C). This suggests that in polyploid *Malus* species, the *RGF* family may influence overall expression dosage by increasing gene copy number, thereby enhancing adaptive capacity to variable environments [31]. Small peptides typically exhibit diverse expression patterns across different organs and tissues [32]. To further elucidate the potential biological functions of the *MdRGF* gene family in ‘Gala’, we investigated their spatial expression patterns across different organs. Using quantitative reverse-transcription PCR (qRT-PCR), we analyzed the expression levels of nine *MdglRGF* genes in the root, stem, leaf, flower, and fruit of apple (Fig. S8). Our analysis revealed that all examined *MdglRGF* genes are transcribed in various tissues but exhibit distinct and tissue-preferential expression profiles, suggesting extensive functional diversification. A subset of genes displayed highly root-predominant expression. Most notably, *MdglRGF1* and *MdglRGF6* exhibited an exceptionally strong root-specific pattern, with their transcript abundance in roots vastly exceeding (by orders of magnitude) the minimal levels detected in any aerial organ. This stark contrast suggests that these genes may play specialized roles in root development or physiology.

To clarify the effect of the *RGF* gene family on root systems within the genus *Malus*, we selected the *RGF* subfamily encoding the mature peptide ‘DYTPARKKPPIHN’—which exhibits high family number and the highest expression level in roots of apple—for further analysis. We found that in *Malus*, the precursor peptides of this type can be divided into two classes based on length. Sequence alignment revealed that the protein sequences within each class are highly conserved, with differences only in single amino acids within the signal peptide length, variable region conserved sequences, and the DY mature motif region (Fig. 3D). In the triploid species *M. transitoria*, the number of *RGF* genes encoding the ‘DYTPARKKPPIHN’ mature peptide reached four, which may be related to its previously reported enhanced environmental adaptability [33]. Subsequently, we analyzed the expression patterns of the *RGF* gene family by integrating root organ transcriptome data from *M. domestica* ‘Gala’. We found significant variation in root expression of *RGF* genes among different individuals at the same developmental stage (Fig. S9A). Chromosomal mapping revealed that *MdglRGF* genes are broadly distributed across multiple chromosomes (chr1, chr2, chr4, chr7, chr13, chr15, chr18) of the ‘Gala’ genome, exhibiting a scattered genomic arrangement. Among these, *MdglRGF5* and *MdglRGF7* are physically clustered in close proximity on chr13 (Fig. S9B), while the remaining members (*MdglRGF1* on chr1, *MdglRGF2.2/MdglRGF3.2* on chr2, *MdglRGF4* on chr4) are individually localized to distinct chromosomal regions without adjacent paralogs. This distribution pattern suggests that *MdglRGF* genes primarily evolved via segmental duplication or transposition events, with only a single tandem duplication event contributing to the *MdglRGF5/MdglRGF7* cluster. Promoter regions play a decisive role in regulating gene transcriptional activity. To better understand the regulatory mechanisms underlying *RGF* gene expression, we performed a detailed *cis*-regulatory element analysis for different *RGF* genes within the same species and for *RGF* families encoding the same mature peptide across different *Malus* species (Fig. 3E). We identified several *cis*-regulatory elements in the promoter regions of *RGF* genes that respond to various hormonal and environmental signals, including Abscisic acid, Anaerobic, Anoxic, Auxin, Cell cycle, Circadian, Endosperm, Gibberellin, Low temperature, MeJA, Meristem, Mixed stress, and MYB Binding Sites. Interestingly, significant differences exist in the promoter cis-elements of the *RGF* genes encoding the ‘DYTPARKKPPIHN’ mature peptide across different varieties (Fig. S10). Unique *cis*-elements associated with seed-specific regulation were found in *M. ioensis* and *M. baccata*, while elements related to zein metabolism regulation appeared in *M. ioensis*, *M. baccata*, and *M. doumeri*. This suggests that in different species, genes encoding the same RGF mature peptide may be activated by distinct regulatory signals. Consistent with prior research, the divergence in *cis*-regulatory elements controlling *RGF* genes may have contributed to the evolutionary changes within the genus *Malus* [34]. Previous studies have reported that members of the LRR-RK subfamily may function as receptors for RGF peptides. To investigate the conservation of RGF receptors in apple, we identified members of the LRR-RK subfamily in the cultivated apple ‘Gala’ based on studies of LRR-RKs and their ligands in *Arabidopsis*, using BLAST homology alignment and manual curation. We found that the number of LRR-RK subfamily members in apple is comparable to that in *Arabidopsis*, and the structures of MdglRGFR1 and MdglRGF1 showed no significant differences compared to their *Arabidopsis* homologs, with the core domains being highly conserved. Molecular dynamics simulations demonstrated that the ‘DYTPARKKPPIHN’ peptide encoded by MdglRGF1 could stably embed into the binding pocket of the MdRGFR1 protein, forming a multi-point interaction network (Fig. S16).

### Sulfation enhances the activity of exogenously applied MdglRGF1 regulating root growth

**Figure 4:**
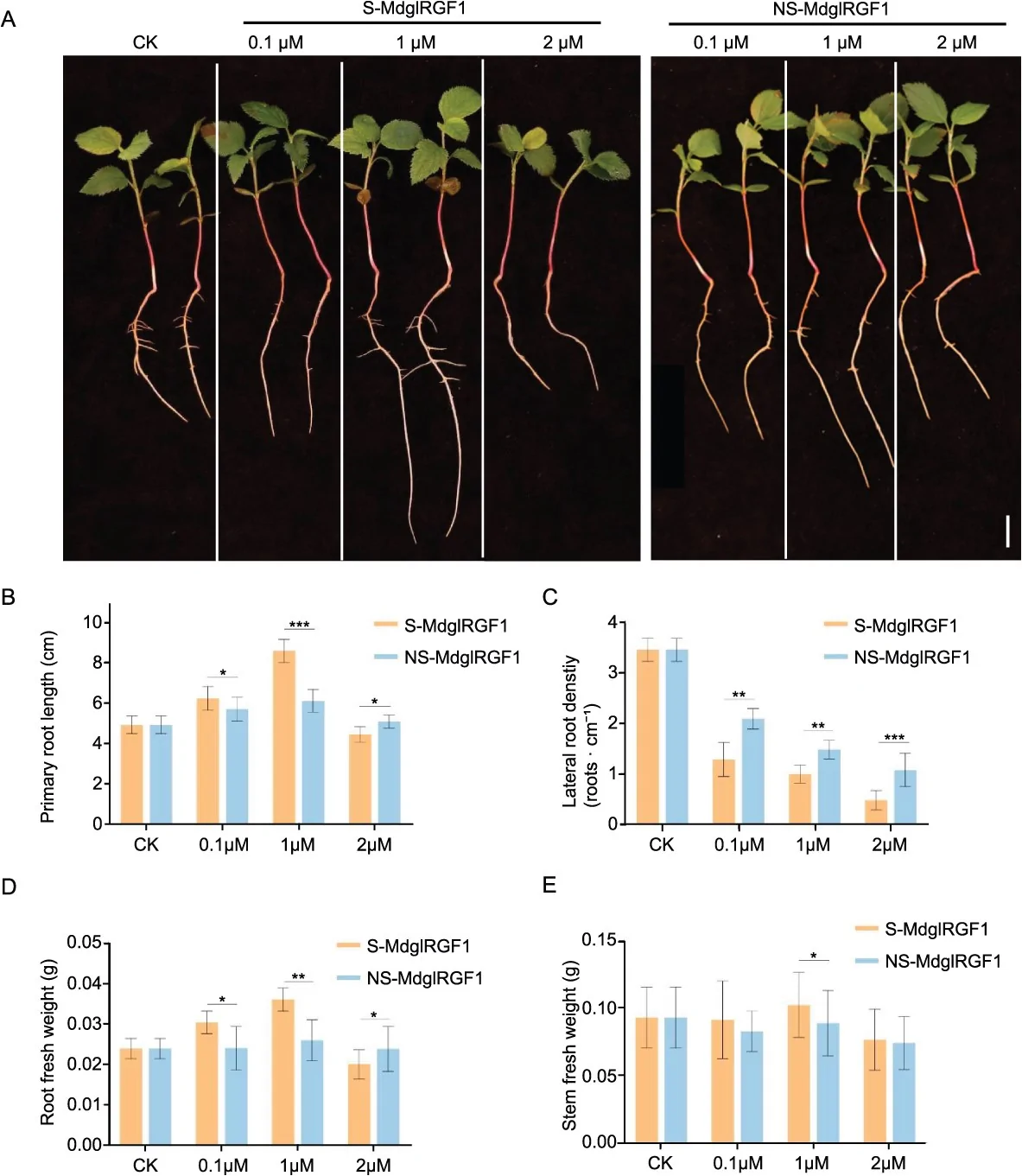
Effects of sulfated and non-sulfated modifications on root development of *M. hupehensis* var. *pingyiensis.* (A) Phenotypic analysis of *M. hupehensis* var. *pingyiensis* seedlings after 25 days of treatment with sulfated (S-MdglRGF1) or nonsulfated (NS-MdglRGF1) MdglRGF1 peptide. Seedlings were treated with different concentrations (0.1, 1, 2 μM) of S-MdglRGF1 or NS-MdglRGF1; untreated seedlings served as the control (CK). Representative images show changes in root system architecture under each treatment. (Bar = 1 cm) (B) Primary root length of *M. hupehensis* var. *pingyiensis* seedlings under S-MdglRGF1/NS-MdglRGF1 treatment. Quantification of primary root length reveals a dual-effect (hormone-like biphasic response) of MdglRGF1 on primary root growth; the regulatory potency of S-MdglRGF1 is significantly stronger than that of NS-MdglRGF1.(C) Lateral root density of *M. hupehensis* var. *pingyiensis* seedlings under S-MdglRGF1/NS-MdglRGF1 treatment. Lateral root density exhibits a concentration-dependent negative response to both peptides, with S-MdglRGF1 inducing a more pronounced inhibitory effect. (D, E) Root and stem fresh weight of *M. hupehensis* var. *pingyiensis* seedlings under S-MdglRGF1/NS-MdglRGF1 treatment. Fresh weight of roots (D) and stems (E) shows differential responses to peptide treatments; S-MdglRGF1 exerts a stronger regulatory effect on biomass accumulation compared to NS-MdglRGF1. Data are presented as mean ± SD (*n* ≥ 6). Statistical significance: *P* < 0.05(^*^), *P* < 0.01(^**^), *P* < 0.001(^***^).

Modulating specific root architectural traits, such as root angle and lateral root density, has been shown to improve plant anchorage and nutrient acquisition under varying environmental conditions [35–37], a trait of substantial relevance to apple production. To explore the potential of plant peptide hormones in regulating apple root development, we employed the apomictic apple rootstock ‘*M. hupehensis* var. *pingyiensis’* as plant material and examined the effects of exogenous MdglRGF1 on seedling root development. We found that exogenous treatment significantly affected root gravitropism in *M. hupehensis*, with a biphasic dose–response pattern, a hallmark of classical plant hormones (Fig. 4A and B). In contrast, lateral root density showed a dose-dependent inhibitory response to the same concentration gradient of the applied peptide (Fig. 4A and C). To evaluate the functional contribution of tyrosine sulfation, we additionally examined the unmodified, non-sulfated MdglRGF1 peptide (NS-MdglRGF1). Notably, the unmodified peptide exhibited the same qualitative regulatory effects as the mature, sulfated peptide when applied exogenously, but its regulatory efficacy was significantly reduced (Fig. 4A–C). To assess whether this regulatory mechanism is species-specific, we supplemented the treatment with a submicromolar concentration (0.01 μM) of the MdglRGF1 peptide and applied the same concentration gradient to the Brassicaceae model plant *A. thaliana*. The results were highly consistent with those observed in apple seedlings (Fig. S11A–C), demonstrating that this mode of root architectural regulation is conserved across species. Furthermore, the application of the sulfated MdglRGF1 peptide enhanced the overall regulatory efficacy of MdglRGF1.

### Overexpression of *MdglRGF1* inhibits adventitious root regeneration during leaf-derived root formation

**Figure 5:**
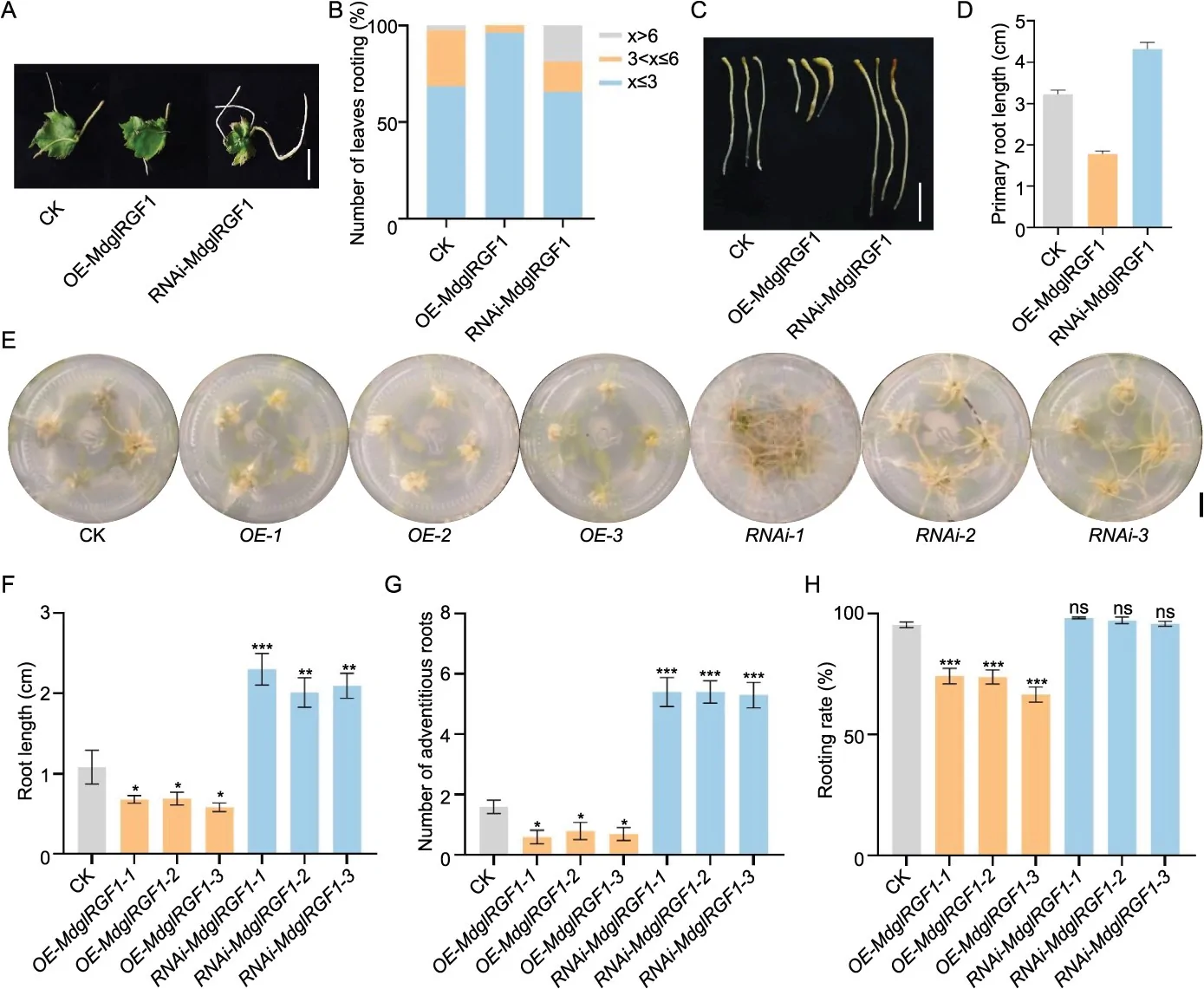
Overexpression of *MdglRGF1* inhibits adventitious root regeneration in the leaf-derived roots (LDRs) of *M. domestica* ‘Gala’. (A) Adventitious root regeneration phenotypes of control (CK), MdglRGF1-overexpressing (*OE-MdglRGF1*), and MdglRGF1-silenced (*RNAi-MdglRGF1*) leaf explants at 30 days after culture (Bar = 1 cm); (B) Percentage of rooted leaves in different lines during leaf-derived roots. x represents the number of roots per single leaf. (C) Morphology of adventitious roots regenerated from different lines at 30 days after culture (Bar = 1 cm). (D) Primary root length of adventitious roots regenerated from different lines. (E) Plate phenotypes of leaf regeneration in different lines at 40 days after culture during rooting induction (Bar = 1 cm). (F) Root length of regenerated adventitious roots in different lines. (G) Number of adventitious roots regenerated from different lines. (H) Rooting rate of regenerated adventitious roots in different lines. The rooting rate was significantly reduced in OE lines compared to CK, while no significant difference was observed in RNAi lines (ns = no statistical significance). Data are presented as mean ± SD (*n* ≥ 3). Statistical significance: *P* < 0.05(^*^), *P* < 0.01(^**^), *P* < 0.001(^***^).

Leaves are commonly used as explants for bud regeneration in *Malus* species [38]. To investigate the effect of *MdglRGF1* on adventitious root formation in apple leaves, we constructed *MdglRGF1* overexpression constructs and RNA interference (RNAi) vectors (Fig. S12A). These constructs were introduced into *Agrobacterium rhizogenes* and subsequently used to infect leaves of the apple cultivar ‘Gala’, resulting in stable transgenic lines with altered *MdglRGF1* expression (Fig. S12B–E). We then used an *A. rhizogenes*–mediated system to quantify the number and length of adventitious roots produced during leaf-derived rooting. Compared with control lines, *MdglRGF1*-overexpressing lines produced significantly fewer and shorter adventitious roots, whereas *MdglRGF1*-silencing lines exhibited the opposite phenotype (Fig. 5A–D). During rooting induction, both the rooting rate and the number of adventitious roots were significantly reduced in *MdglRGF1*-overexpressing lines. In contrast, *MdglRGF1*-silencing lines exhibited a significant increase in the number and length of adventitious roots (Fig. 5E–H).

### Overexpression of *MdglRGF1* negatively regulates adventitious root growth and development in apple seedlings

**Figure 6:**
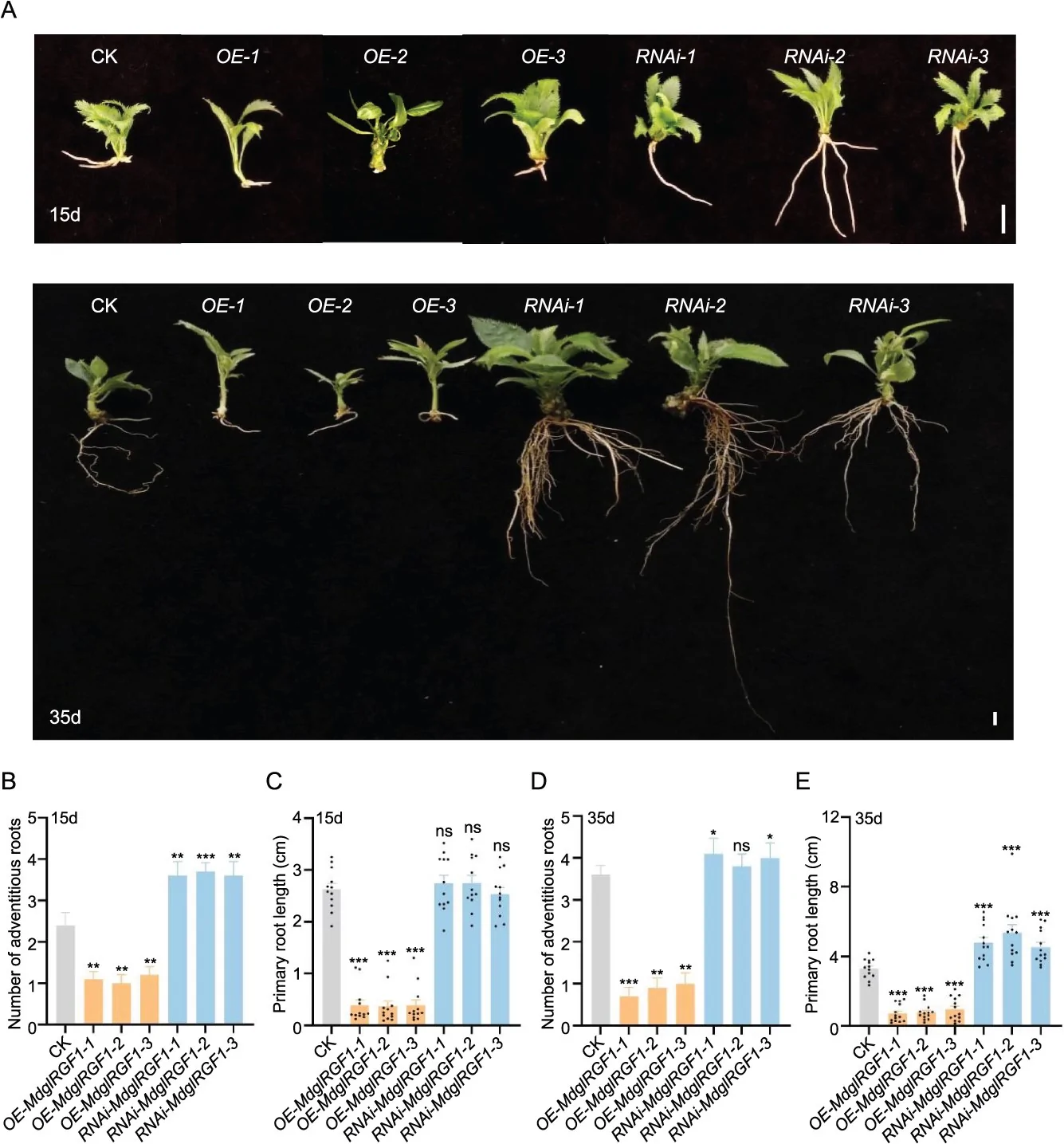
*MdglRGF1* overexpression negatively regulates the growth and development of *M. domestica* ‘Gala’ seedlings. (A) Phenotypes of control (CK), *MdglRGF1*-overexpressing (*OE-MdglRGF1*), and *MdglRGF1*-silenced (*RNAi-MdglRGF1*) seedlings at 15 and 35 days postcutting propagation. Representative images show seedling growth (including shoot and root architecture) under soil culture conditions (Bar = 1 cm). (B) Number of adventitious roots in seedlings at 15 days postcutting. Quantification results demonstrate that OE lines produced almost no adventitious roots, while RNAi lines exhibited vigorous root growth compared to CK. (C) Primary root length of seedlings at 15 days postcutting. Primary root length was significantly shorter in OE lines, whereas RNAi lines showed longer primary roots relative to CK (ns = no statistical significance). (D) Number of adventitious roots in seedlings at 35 days postcutting. OE lines had fewer adventitious roots than CK, while RNAi lines displayed a significant increase in adventitious root number. (E) Primary root length of seedlings at 35 days postcutting. Primary root length was reduced in OE lines and significantly elongated in RNAi lines compared to CK. Data are presented as mean ± SD (*n* ≥ 6). Statistical significance: *P* < 0.05(^*^), *P* < 0.01(^**^), *P* < 0.001(^***^).

To assess the role of the MdglRGF1-encoded peptide hormone in apple seedling growth and development, we cultivated the transgenic lines and control plants in soil via vegetative cuttings. After 15 days of cultivation, *MdglRGF1*-overexpressing (*OE-MdglRGF1*) lines showed severely impaired root formation compared with the control, whereas the MdglRGF1-silenced (*RNAi-MdglRGF1*) lines exhibited the opposite phenotype, with vigorous root growth. By 35 days after planting, the *MdglRGF1*-overexpressing lines produced fewer adventitious roots and shorter primary roots relative to the control (Fig. 6A–C). In contrast, the silencing lines showed a significant increase in the number of roots and significant elongation of the primary root (Fig. 6D and E). These observations are consistent with the results obtained from the rooting induction of transgenic plants in tissue culture.

**Figure 7:**
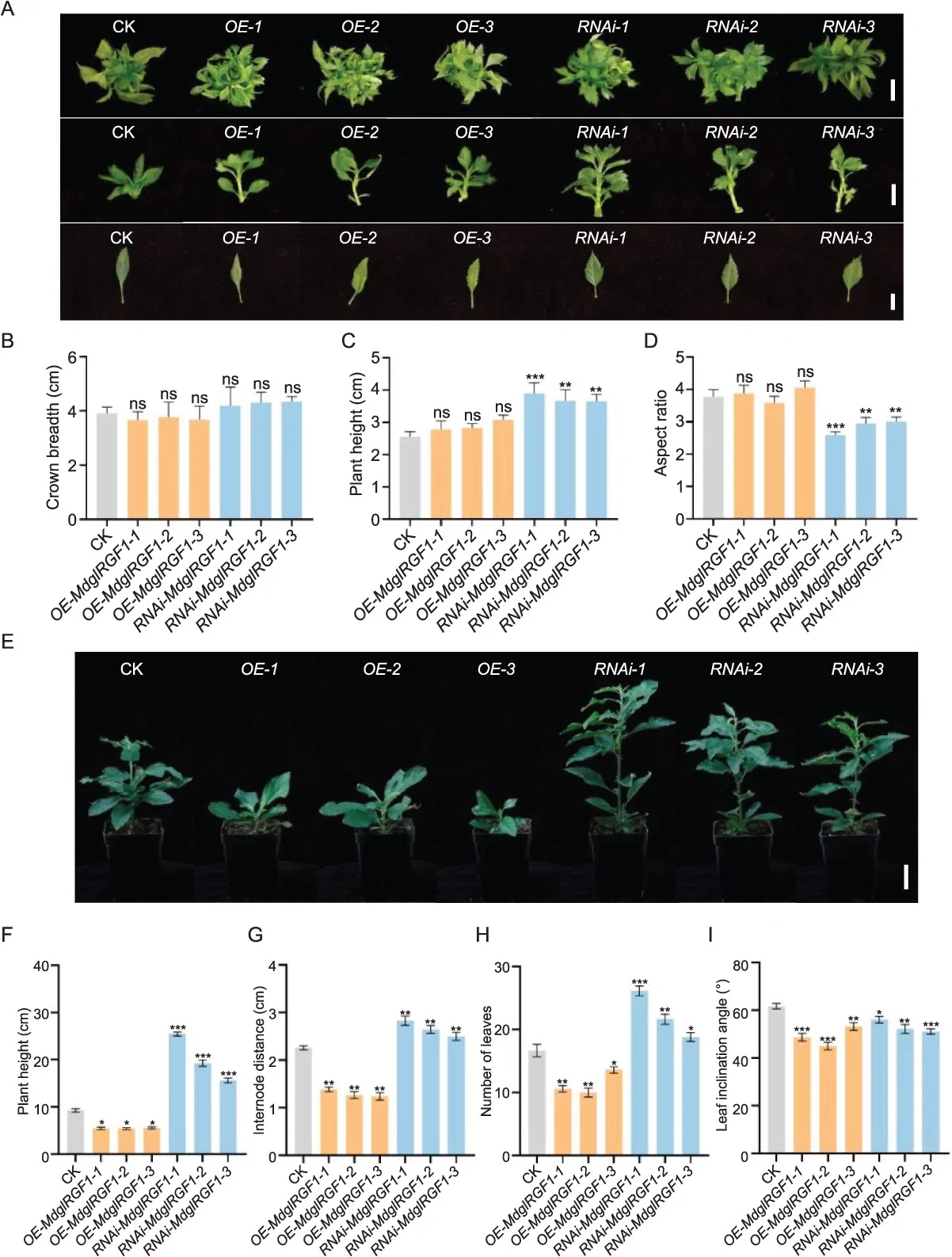
*MdglRGF1* negatively regulates the aboveground growth and development of *M. domestica* ‘Gala’ seedlings. (A) Phenotypes of *in vitro*–cultured seedlings (30 days, nonrooted) in control (CK), *MdglRGF1*-overexpressing (*OE-MdglRGF1*), and *MdglRGF1*-silenced (*RNAi-MdglRGF1*) lines. Representative images show crown, leaf, and shoot architecture of nonrooted seedlings; scale bars indicate the size of samples (Bar = 1 cm). (B–D) Quantification of morphological traits of 30 days *in vitro*–cultured seedlings. (B) Crown breadth. (C) Plant height. (D) Leaf aspect ratio. (E) Phenotypes of soil-cultured seedlings (90 days postcutting) in different lines. Representative images display the overall aboveground growth status (Bar = 5 cm). (F–I) Quantification of aboveground traits in 90-day soil-cultured seedlings. (F) Plant height. (G) Internode distance. (H) Number of leaves. (I) Leaf inclination angle. Data are presented as mean ± SD (*n* ≥ 6). Statistical significance: *P* < 0.05(^*^), *P* < 0.01(^**^), *P* < 0.001(^***^).

To explore the molecular basis underlying the inhibitory effect of *MdglRGF1* overexpression on adventitious root formation in apple seedlings, we conducted transcriptome sequencing of 180-day-old ‘Gala’ roots. Differential expression analysis revealed 1714 upregulated and 2187 downregulated genes (Table S9). GO enrichment analysis of these differentially expressed genes indicated significant associations with processes such as ‘response to stimulus’, ‘response to stress’, and ‘regulation of phosphorus metabolic process’ (Table S10). This suggests that *MdglRGF1* overexpression may indirectly influence adventitious root development through alterations in metabolic and nutrient-related pathways (Fig. S13). To further assess the impact of *MdglRGF1* on shoot growth and development, we measured a suite of phenotypic parameters in 30-day-old transgenic plantlets under rootless conditions in culture, including canopy diameter, plant height, leaf length-to-width ratio, stem height, stem width, leaf width, leaf number, and propagation coefficient (Fig. 7A and B; Fig. S14). Compared to the control, *MdglRGF1*-silencing lines showed a significant increase in plant height and a significant decrease in leaf length-to-width ratio, resulting in increased plant height and altered leaf morphology (Fig. 7C and D). Subsequently, after 90 days of soil cultivation via cuttings, *MdglRGF1*-overexpressing lines exhibited slower shoot growth, shorter stature, reduced internode length, and fewer leaves. In contrast, *MdglRGF1*-silencing lines displayed faster shoot growth, significantly increased plant height, longer internodes, and more leaves (Fig. 7E–I). Both overexpression and silencing of *MdglRGF1* significantly reduced the leaf angle of apple seedlings. Collectively, these results demonstrate that *MdglRGF1* functions as a negative regulator of both adventitious root formation and shoot development, exerting pleiotropic effects on apple seedling architecture.

### *MdglRGF1* regulates adventitious root development by coordinating auxin biosynthesis and polar transport

**Figure 8:**
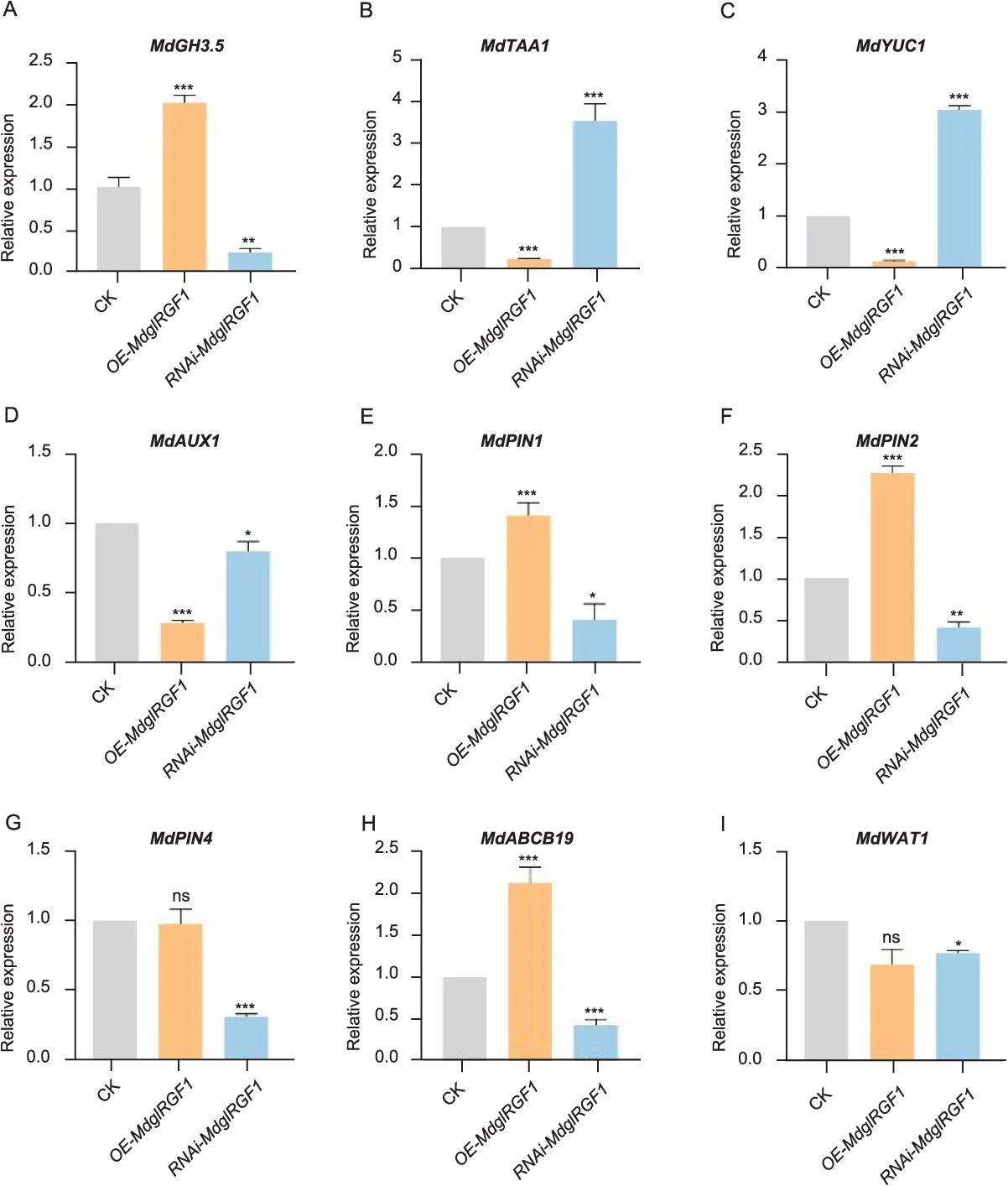
RT-qPCR analysis of auxin pathway-related gene expression in *MdglRGF1* transgenic apple adventitious roots after 30 days of rooting culture. (A) *MdGH3.5* (auxin marker), (B, C) auxin biosynthesis genes (*MdTAA1*, *MdYUC1*), and (D–I) auxin transporter genes (*MdAUX1*, *MdPIN1*, *MdPIN2*, *MdPIN4*, *MdABCB19*, *MdWAT1*), with relative expression levels shown for empty vector control (CK), *MdglRGF1* overexpression (*OE-MdglRGF1*), and *MdglRGF1* RNA interference (*RNAi-MdglRGF1*) lines; significance denoted as *P* < 0.05 (^*^), *P* < 0.01 (^**^), *P* < 0.001 (^**^^*^), and nonsignificant (ns).

Combined with the preceding findings, we propose that the influence of *MdglRGF1* on adventitious root development in apple is likely associated with the auxin signaling pathway. To further dissect the mechanism by which *MdglRGF1* negatively regulates apple adventitious rooting, we quantified auxin-related gene expression via RT-qPCR in transgenic adventitious roots: *MdglGH3.5* and *MdglLBD29* showed diametrically opposed patterns (significantly upregulated in *OE-MdglRGF1* vs. downregulated in *RNAi-MdglRGF1* relative to the control, CK), *MdglIAA19* and *MdglLBD16* were unaltered in *OE-MdglRGF1* but reduced in *RNAi-MdglRGF1* (linking *MdglRGF1* to auxin signaling), while profiling of auxin pathway genes (Fig. 8; Fig. S15A–C) revealed that *OE-MdglRGF1* roots exhibited downregulated *MdglAUX1* (influx transporter) and biosynthesis genes (*MdglTAA1*, *MdglYUC1*) alongside upregulated efflux transporters (*MdglPIN1*, *MdglPIN2*, *MdglABCB19*), and *RNAi-MdglRGF1* roots showed reduced efflux transporters (*MdglPIN1*, *MdglPIN2*, *MdglPIN4*, *MdglABCB19*) and elevated *MdglTAA1*/*MdglYUC1*. Notably, the expression levels of *MdglLBD29* and *MdglLBD16* were lower in the RNAi-*MdglRGF1* lines than in the OE lines. To determine whether these altered expression patterns were associated with developmental timing, we examined the transcript levels of adventitious root-related genes during the early stage of adventitious root development (Fig. S15D). The results showed that, compared with those in OE-*MdglRGF1* plants, the expression levels of *MdglLBD29*, *MdglLBD16*, *MdglARF6*, *MdglARF8*, *MdglWOX11*, and *MdglRGI1* were all downregulated in RNAi-*MdglRGF1* plants. These results suggest that in OE-*MdglRGF1* plants, sustained RGF signaling maintains roots at the ‘initiation’ stage, with continuous high expression of related genes, while excessive auxin biosynthesis inhibits adventitious root formation and outgrowth. Together, these expression patterns suggest that MdglRGF1 modulates adventitious root development by repressing auxin biosynthesis and enhancing auxin efflux to alter auxin homeostasis.

## Discussion

In graft-propagated crops, the rootstock root system plays crucial roles in nutrient and water uptake, stress tolerance enhancement, and overall plant resilience [39, 40]. In contemporary root biology research, certain small secreted peptides exhibit extensive and diverse functions in apple. For instance, the IDA peptide regulates lateral root growth in this species [41], while RGF peptides are recognized as key signaling molecules mediating plant development, particularly root patterning [7, 8, 23]. In this study, we constructed a small secreted peptide library encompassing 64 plant species, covering a broad phylogenetic spectrum. The quantity of small secreted peptides in plants showed a positive correlation with genome size. Notably, the number of signal peptides in *Malus × platycarpa* shows an opposite trend compared with other *Malus* species. We speculate that this phenomenon may be related to its polyploid characteristics; however, due to the lack of in-depth studies and direct supporting evidence, this is presented only as a hypothesis. Since whole-genome duplication (WGD) events increase plant genome size [42], they consequently augment the repertoire of small secreted peptides, which may contribute to enhanced adaptability to complex and variable environments. Subsequently, we annotated small secreted peptides using the root transcriptome of the cultivated apple *M. domestica* ‘Gala’. We found that the expression of these small secreted peptides varies substantially among individuals at the same developmental stage. This dynamic regulation likely facilitates the formation of precise signaling networks among different peptides [43, 44], enabling coordinated responses to environmental challenges. Such dynamic regulation of small secreted peptides has been demonstrated across various plant tissues and organs, including the inflorescence of Asteraceae species [45], different tissues in maize [46], roots of *Arabidopsis* [47], and fruits of tomato [48]. We then performed genome-wide identification and phylogenetic analysis of RGF peptides across the 64 plant species. In the bryophytes *A. angustus* and *B. argenteum*, we identified RGF-like peptides containing the conserved DY motif, consistent with previous findings that canonical RGF peptides emerged after the evolution of true roots [49]. In our study, the *RGF* gene family, characterized by a complete secretory signal peptide and the DY motif, underwent a dramatic expansion in ferns. This expansion may be linked to multiple independent WGD events within fern genomes and the significant morphological diversification of ferns [50–52], which conferred environmental adaptability to withstand various stresses [53, 54]. During subsequent plant evolution, the *RGF* family experienced contraction, followed by a new round of expansion associated with polyploidization events in angiosperms. Within the genus *Malus*, we identified several clusters of *RGF* genes encoding identical mature peptides. Amino acid sequences within the same cluster were highly conserved. We further observed that the promoters of genes encoding the same mature RGF peptide in different *Malus* accessions harbored distinct *cis*-regulatory elements, potentially enhancing the responsiveness of RGF signaling to diverse environmental cues.

Ideal root system architecture is a complex trait controlled by multiple genetic and environmental factors [55]. In rice, two genes have been identified as major contributors to variation in ideal root phenotypes. One representative example is *DRO1* [56], which regulates root gravitropism leading to steeper growth angles and deeper roots, fitting the ‘Steep, Cheap and Deep’ ideotype [57]. In *Arabidopsis*, AtRGF1 is sulfated by TPST and perceived by RGIs/RGFRs to regulate root apical meristem development. Our study investigated the effect of exogenous application of RGF peptides on apple root systems. We found that sulfation significantly enhanced the regulatory efficacy of the applied peptide compared to its nonsulfated form, mirroring the importance of post-translational modification for peptide hormone activity *in planta* [58, 59]. Furthermore, for certain root traits like primary root length, exogenous peptide application exhibited a clear dose-dependent effect. In practical production, applying RGF peptides at optimal concentrations could be a strategy to modulate rootstock root architecture toward a ‘steep and deep’ ideotype. By virtue of the characteristic that seedling traits partially reflect the phenotypic features of mature plants, supporting ‘deep rooting’ breeding for improved drought tolerance in apple rootstocks [60].

Through gain and loss-of-function analyses of *MdglRGF1* (a gene encoding the mature peptide ‘DYTPARKKPPIHN’) in *M. domestica* ‘Gala’, including RNAi approaches, we found that overexpression of *MdglRGF1* significantly inhibited the regeneration and growth of adventitious roots during plant growth and leaf-based regeneration. Additionally, *MdglRGF1* affected shoot development under rootless conditions. The regulation of apple growth and development by *MdglRGF1* exhibits organ-specificity and spatiotemporal dependence, providing a new perspective for deciphering the complex growth regulatory networks in fruit trees. In root development, *MdglRGF1* influenced root architecture by negatively regulating adventitious root number and primary root length. Transcriptome analysis revealed that differentially expressed genes (DEGs) were enriched in pathways such as ‘response to stimulus’ and ‘regulation of phosphorus metabolic process’, suggesting that *MdglRGF1* may modulate root development by integrating environmental signals and nutrient metabolism processes. This regulatory mechanism of root nutrient development is analogous to the functions of certain microRNAs [61]. This regulatory mode resembles that reported for C-terminally encoded peptides (CEPs) peptides, which synergistically regulate root growth with the cytokinin pathway via CEP DOWNSTREAM (CEPD) glutaredoxins [62], reflecting the crosstalk characteristics between plant peptide hormones and other signaling pathways [63].

In this study, both exogenous application of the MdglRGF1 peptide and RNAi-mediated silencing of *MdglRGF1* promoted plant growth, reflecting the complexity and spatiotemporal specificity of RGF family peptide signaling. Exogenous MdglRGF1 treatment exhibited a typical concentration-dependent biphasic effect, in which an appropriate concentration promoted primary root growth, whereas a high concentration induced signal saturation or antagonistic effects. This represents a common regulatory pattern of plant peptide hormones. In contrast, transient exogenous application and RNAi-mediated chronic genetic silencing differ fundamentally. The former represents short-term acute signal regulation affecting primary root growth at the seedling stage, whereas the latter may suppress endogenous gene expression throughout the entire developmental period, thereby triggering compensatory pathways and metabolic reprogramming, which, in turn, promote overall plant growth. Therefore, we speculate that the regulation of plant growth by *MdglRGF1* may be mediated by multilayered and fine-tuned control jointly determined by treatment mode, effective concentration, and developmental stage, which is consistent with the complex regulatory mechanisms of the *RGF* family in land plants.

We hypothesize that apple may possess an adventitious root development pathway distinct from that characterized in *A. thaliana*. This alternative pathway appears to operate independently of the previously reported adventitious rooting module. Upon suppression of MdglRGF1 expression, apple likely initiates adventitious root formation through a novel regulatory route. Studies in Arabidopsis have demonstrated widespread functional redundancy and differentiation among RGF family members, such that silencing individual RGF genes typically does not alter root system architecture. In contrast, our data reveal that silencing MdglRGF1 alone in ‘Gala’ apple produces a pronounced adventitious root phenotype. This finding suggests that the peptide encoded by MdglRGF1 may function as a central signaling component in a pathway that plays a decisive role in adventitious root development in apple.

In shoot growth, *MdglRGF1*-silenced lines showed increased plant height and rounder leaves at the in vitro culture stage. After 90 days of soil cultivation, these lines exhibited longer internodes and more leaves, while the growth differences among all lines diminished after 180 days. This indicates that the regulatory effect of *MdglRGF1* is mainly concentrated in the early seedling stage, and this spatiotemporal specificity may be closely associated with the signal requirements for organogenesis during apple seedling development. Furthermore, both overexpression and silencing of *MdglRGF1* significantly reduced the leaf inclination angle, suggesting that MdglRGF1 may exert context-dependent or asymmetric effects on plant architecture, and its underlying molecular mechanism warrants further investigation.

## Materials and methods

### Plant materials and treatments

The plant materials used in this study, *M. hupehensis* and *M. domestica* ‘Gala’ apple, were maintained long-term in our laboratory. Sterile tissue-cultured seedlings were maintained in a growth chamber under controlled conditions (25°C, 16-h light/8-h dark cycle) for subsequent experiments. Stable transgenic ‘Gala’ apple lines were also propagated as sterile tissue-cultured seedlings under identical growth chamber conditions, with subculturing every 30 days for long-term maintenance.

Seeds were sterilized via a sequential procedure as follows: surface-sterilized with undiluted sodium hypochlorite solution for 7 min, repeated three times sequentially; subsequently disinfected with 75% (v/v) ethanol supplemented with Tween-20 (200 μl Tween-20 per 100 ml ethanol) for 40 s; and rinsed thoroughly with sterile distilled water at least six times. The sterilized seeds of *M. hupehensis* and *A. thaliana* ecotype Columbia (Col-0) were sown evenly on half-strength Murashige and Skoog (1/2 MS) medium, followed by stratification at 4°C in the dark. After germination, the seedlings were transferred to a growth chamber and cultured under conditions of 25°C with a 16-h light/8-h dark photoperiod.

For soil cultivation assays, ‘Gala’ apple and *A. thaliana* Columbia (Col-0) seedlings were transplanted into a mixed substrate consisting of nutrient-rich growth medium and coarse vermiculite at a volume ratio of 1:1. All plants were grown in a growth chamber maintained at 25°C with a 16-h light/8-h dark cycle for subsequent phenotypic analysis.

### Construction of a pan-species small secreted peptide library

According to the definition of small secreted peptides, we predicted the signal peptide regions and structures across 64 species using SignalP 6.0 [64] with default parameters under the standard command (signalp -batch 30 000 -org -fasta -gff3 -mature), thereby constructing a species-specific signal peptide library. Subsequently, we performed transmembrane domain prediction for all signal peptides in this database using TMHMM 2.0 with default parameters via the standard command (tmhmm --short < A.pep.fa > tmhmm.out) [65]. Signal peptides localized extracellularly and lacking transmembrane domains were manually screened to establish a secreted peptide library. Among them, manual screening involved integrating the results from TMHMM 2.0, specifically retaining polypeptides with ‘Number of predicted TMHs’ equal to 0 (i.e. lacking a transmembrane domain). Concurrently, the transmembrane region prediction map indicated that the signal peptide directs this polypeptide to localize on the extracellular side. Polypeptide chains meeting both of these criteria satisfied the definition of a secreted peptide and were subsequently compiled into the species-specific Secreted Peptide Library. Finally, protein sequences in the secreted peptide library were subjected to sequence alignment and visualization using MEGA 12.0.10 [66], and sequences with lengths ranging from 0 to 300 amino acids were retained to generate a pan-species small secreted peptide library.

### Genome-wide identification and characterization of the root meristem growth factor peptide gene family

Screening of protein sequences in the RGF small peptide database was performed using the Pfam (v32.0) domain: PTHR34961 [67], Panther (v14.1) [68], and Superfamily (v1.75) databases [11]. Additionally, the sequence BLAST method was employed for further validation of the screened protein sequences [69]. Finally, the identified homologous sequences were aligned using MAFFT software with default parameters (parameter -m MFP), allowing the software to automatically test and select the optimal alignment model. Subsequently, a phylogenetic tree was constructed using IQ-TREE, with 1000 iterations set to perform ultra-fast bootstrap analysis and SH-aLRT test. These approaches were used to assess the robustness of the phylogenetic tree.

### Synthesis and application of synthetic plant peptide hormones

All synthetic plant peptide hormones were synthesized by Sangon Biotech Co., Ltd. (Shanghai, China). According to the molecular weight and purity of each peptide, the required volume was calculated, and the peptides were dissolved in 10% (v/v) DMSO to prepare a 1 mM stock solution, which was aliquoted and stored at −80°C. For exogenous treatments, the stock solution was added to the culture medium and diluted to final concentrations of 0, 0.1, 1, and 2 μM.

#### Exogenous peptide treatment in *M. hupehensis* var. *pingyiensis*

Surface-sterilized seeds were placed on agar plates and stratified at 4°C in darkness for 40 days, then transferred to a growth chamber at 22°C under light for 2 days. After germination, seedlings were cultured on 1/2 MS medium for 15 days and uniformly grown seedlings were selected as experimental materials. These seedlings were transferred to medium containing different concentrations of peptide hormones and cultured for an additional 25 days. The culture medium was replaced every 7 days. At the end of the treatment, plant phenotypes were recorded and analyzed.

#### Exogenous peptide treatment in *A. thaliana*

Surface-sterilized *Arabidopsis* seeds were plated on 1/2 MS medium, stratified at 4°C in darkness for 2 days, and then transferred to a growth chamber at 22°C under light for 4 days (until seedling roots reached ~1 cm in length). Under aseptic conditions, seedlings were transferred to medium supplemented with different concentrations of peptide hormones and grown for an additional 10 days at 22°C under light. Plant phenotypes were then recorded and analyzed.

### Generation of transgenic plants

Transgenic *M. domestica* plants were generated via the hairy root-mediated shoot regeneration transformation method [70]. Leaf explants were excised from 30-day-old wild-type (WT) ‘Gala’ seedlings and used as the recipient material for genetic transformation. The procedures for vector construction, genetic transformation, and subsequent gene expression level analysis were performed according to the protocols described in the referenced study.

### Expression pattern analysis

To investigate the tissue-specific expression patterns of the *MdRGF* gene family members, total RNA was isolated from *M. domestica* tissues using the Omini Plant RNA Kit (CWbio, China). Genomic DNA was removed during the RNA extraction process, and first-strand complementary DNA (cDNA) was synthesized using the PrimeScript™ RT Reagent Kit (Takara, Japan). qRT-PCR was performed with SYBR Green Pro Taq HS (Accurate Biotechnology, Taiwan, China) on a CFX96™ Real-Time PCR Detection System (Bio-Rad, Hercules, CA, USA). All gene-specific primers used for qRT-PCR amplification are listed in Table S1.

### Transcriptomic analysis

Transcriptome sequencing (RNA-seq) was conducted by Benagen Co., Ltd. (China). RNA samples extracted independently from both control (CK) and treatment groups were used for cDNA library construction, with three biological replicates set for each group. Differentially expressed genes (DEGs) were identified with the screening criteria of fold change (FC) ≥ 2 and false discovery rate (FDR) < 0.01.

### Statistical analysis

Statistical analyses were performed using GraphPad Prism v.8 or Microsoft Excel 365 (v.16.0). Two-tailed paired *t*-tests were performed when comparing two categories. Significance was indicated as ^*^P-value < 0.05, ^**^P-value < 0.01, ^***^P-value < 0.001, and ‘ns’ means no significant difference. Apart from these, all remaining experiments were independently repeated at least three times, with consistent results.

## Supplementary Material

Web_Material_uhag151

## Data Availability

All data supporting the findings of this study are available in the article, the extended data figures, or the Supplementary information.
