# Supplementary material for: Pan-species genome-wide evolution of the RGF peptide gene family and its impact on adventitious root regeneration in apple
Source: Hortic Res. 2026 Apr 28;13(9):uhag151. doi: 10.1093/hr/uhag151 (PMC13503966; doi:10.1093/hr/uhag151)
Supplement: Supplementary file 1 [file web_material_uhag151.zip › __.pdf]

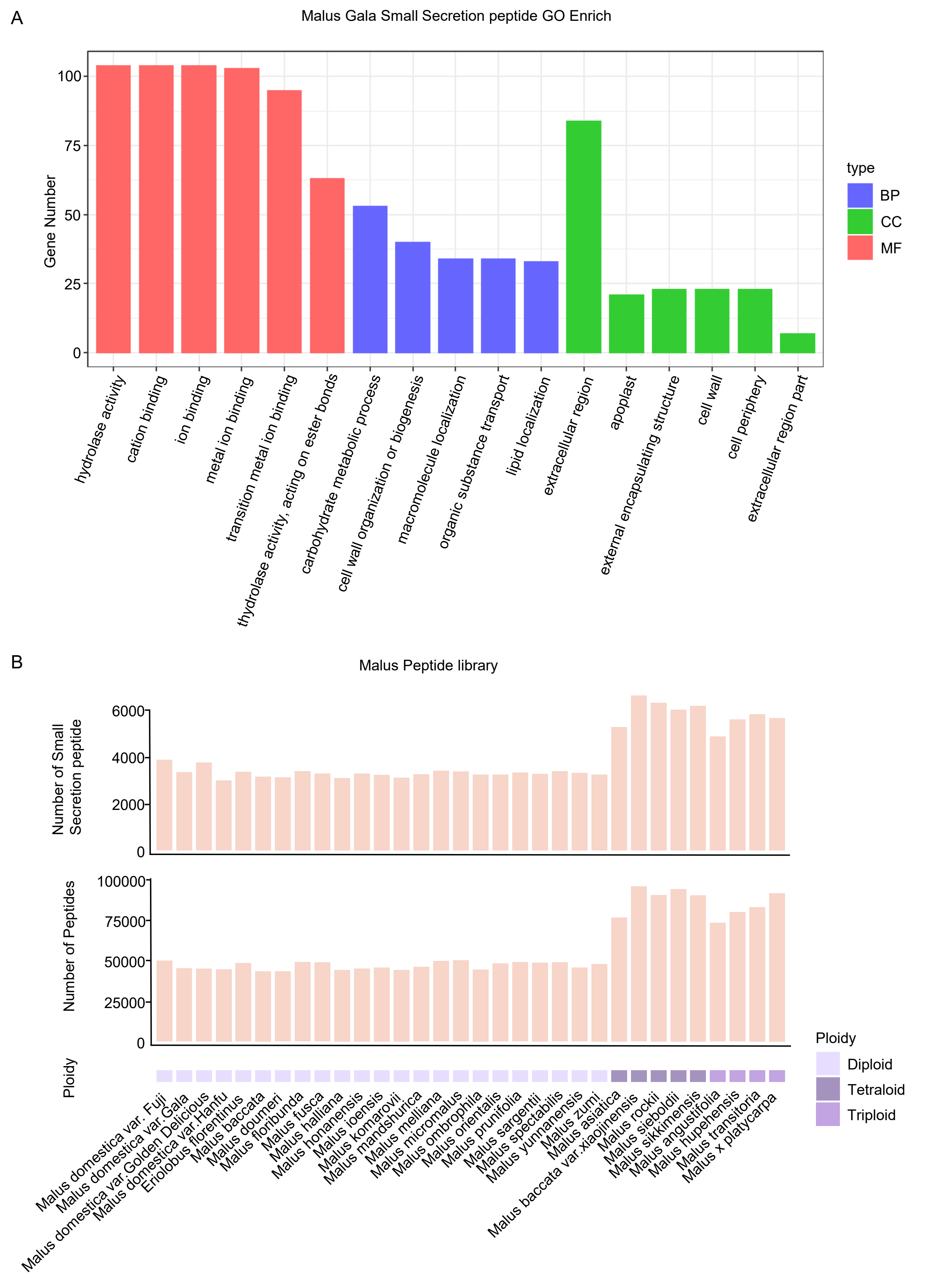

Figure S1. GO enrichment and peptide library characterization of small secreted peptides in *Malus*.(A) Gene Ontology (GO) enrichment analysis of predicted small secreted peptides from Malus × domestica 'Gala'. Bar plot showing the gene count of enriched GO terms, grouped by three GO categories: biological process (BP, blue), cellular component (CC, green), and molecular function (MF, red). (B) Profiling of the Malus peptide library. Upper panel: Number of small secreted peptides across Malus species. Lower panel: Total peptide count per Malus species. Ploidy (diploid, tetraploid, triploid) is annotated for each Malus taxon.

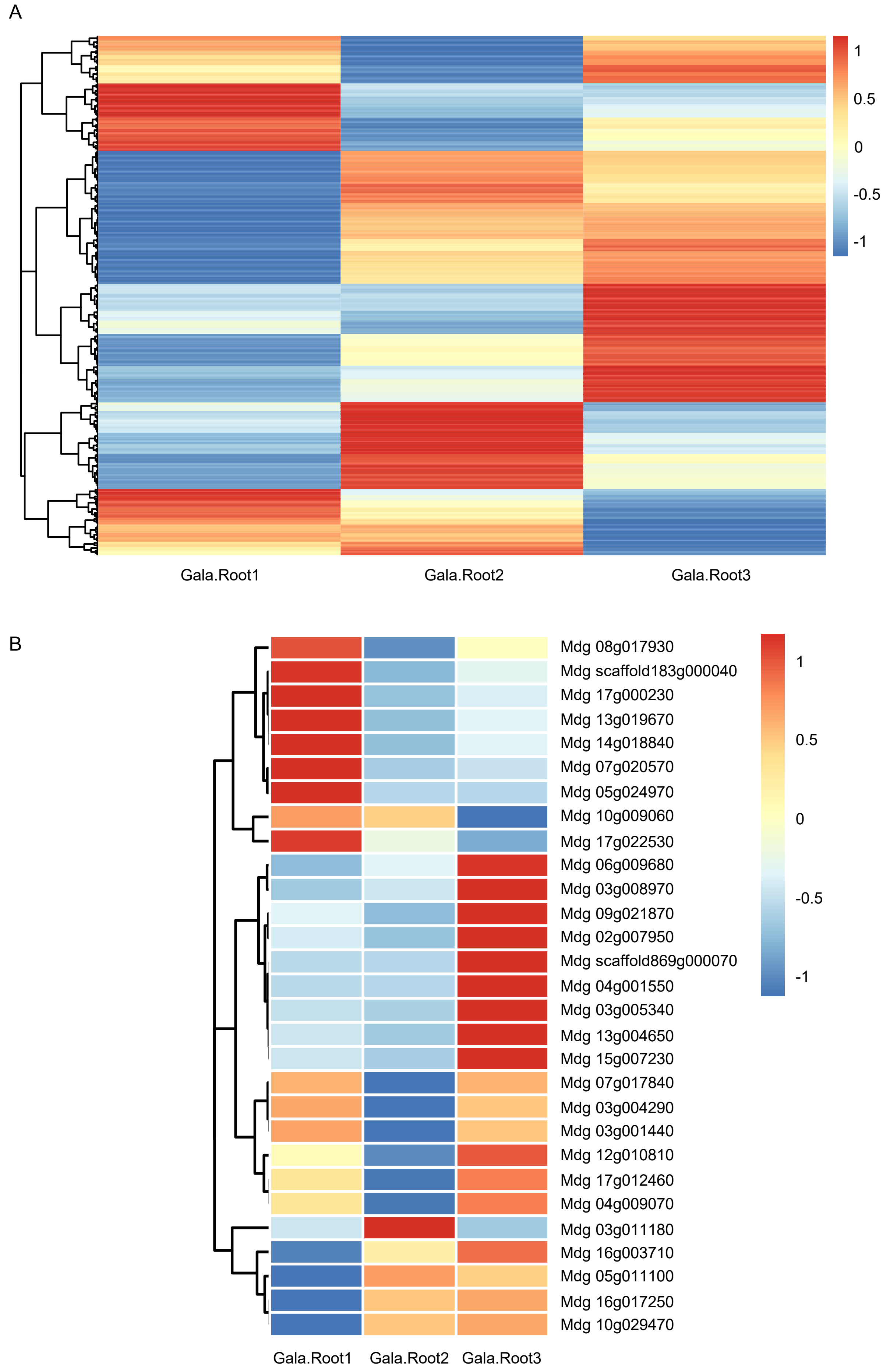

Figure S2. Heatmaps of small secreted peptide expression in *Malus × domestica* ‘Gala’ root tissues.(A) Hierarchical clustering heatmap of expression profiles for all predicted small secreted peptides across three biological replicates (Gala_Root1, Gala_Root2, Gala_Root3) of ‘Gala’ roots; the color gradient (red to blue) denotes normalized expression levels (scaled 1 to -1). (B) Hierarchical clustering heatmap of expression patterns for small secreted peptides stably detected in all three ‘Gala’ root replicates; the color gradient (red to blue) denotes normalized expression levels (scaled 1 to -1).

GO_acc	term_type	Term	pvalue	FDR
GO:0032502	BP	developmental process	0.35	1
GO:0044238	BP	primary metabolic process	0.99	1
GO:0009987	BP	cellular process	0.94	1
GO:0050896	BP	response to stimulus	0.75	1
GO:0048856	BP	anatomical structure development	0.34	1
GO:0044237	BP	cellular metabolic process	0.99	1
GO:0044767	BP	single-organism developmental process	0.33	1
GO:0071704	BP	organic substance metabolic process	0.99	1
GO:0044763	BP	single-organism cellular process	0.88	1
GO:0044699	BP	single-organism process	0.79	1
GO:0008152	BP	metabolic process	1	1
GO:0071840	BP	cellular component organization or biogenesis	0.35	1
GO:0003824	MF	catalytic activity	0.75	1
GO:0005488	MF	binding	0.86	1
GO:0005576	CC	extracellular region	6.10E-07	7.00E-06
GO:0071944	CC	cell periphery	0.023	0.13
GO:0016020	CC	membrane	0.13	0.51
GO:0005623	CC	cell	1	1
GO:0044464	CC	cell part	1	1

Figure S3. Gene Ontology (GO) enrichment analysis of Malus root-specific small secreted peptides.This panel displays the enriched GO terms corresponding to the root-specific small secreted peptides of *Malus*.

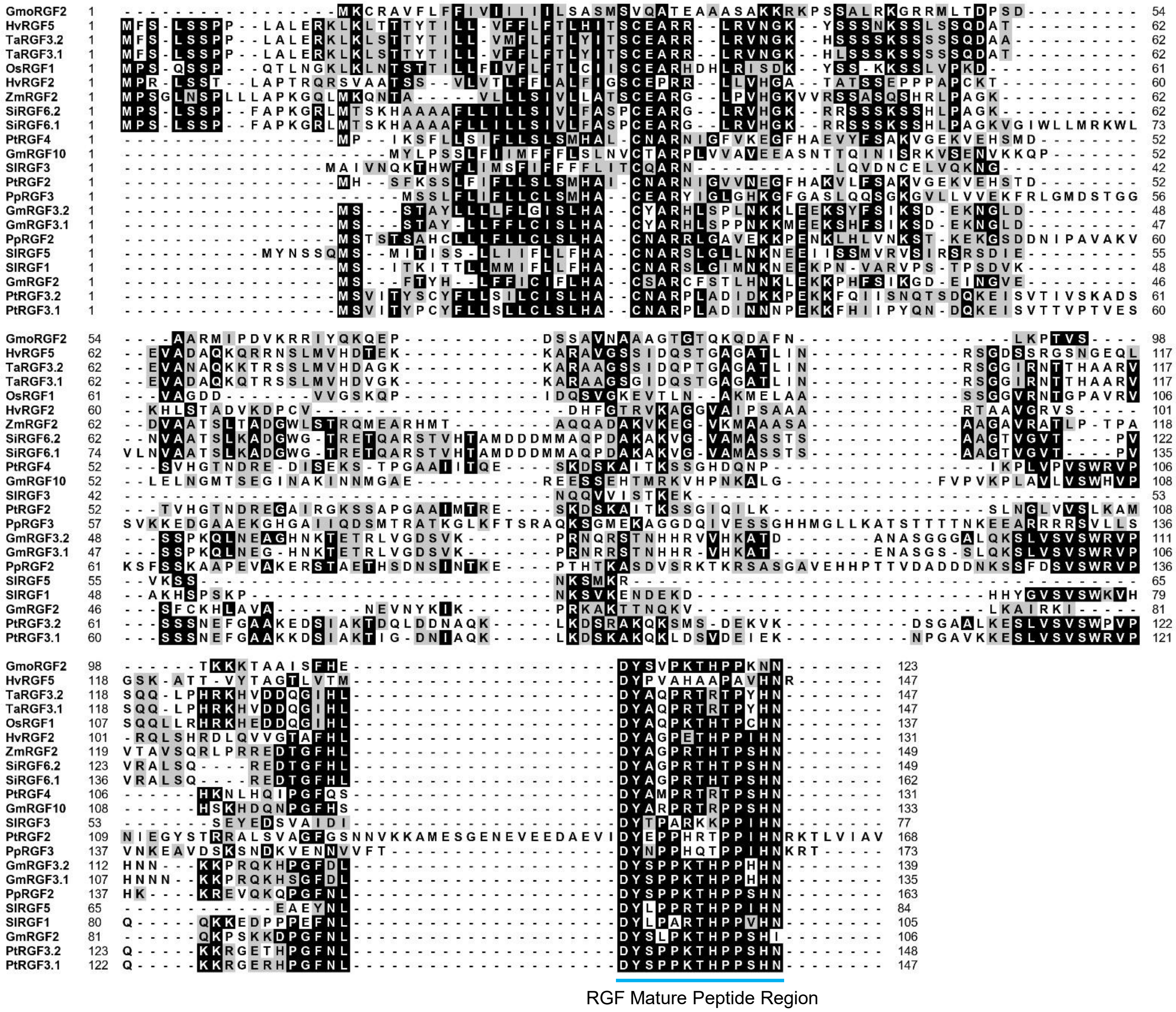

Figure S4. Amino acid sequence alignment of RGF precursor peptides from diverse plant species.This panel displays the sequence alignment of RGF precursor peptides corresponding to RGF gene family members across different plant taxa; the RGF Mature Peptide Region is highlighted by a blue box.

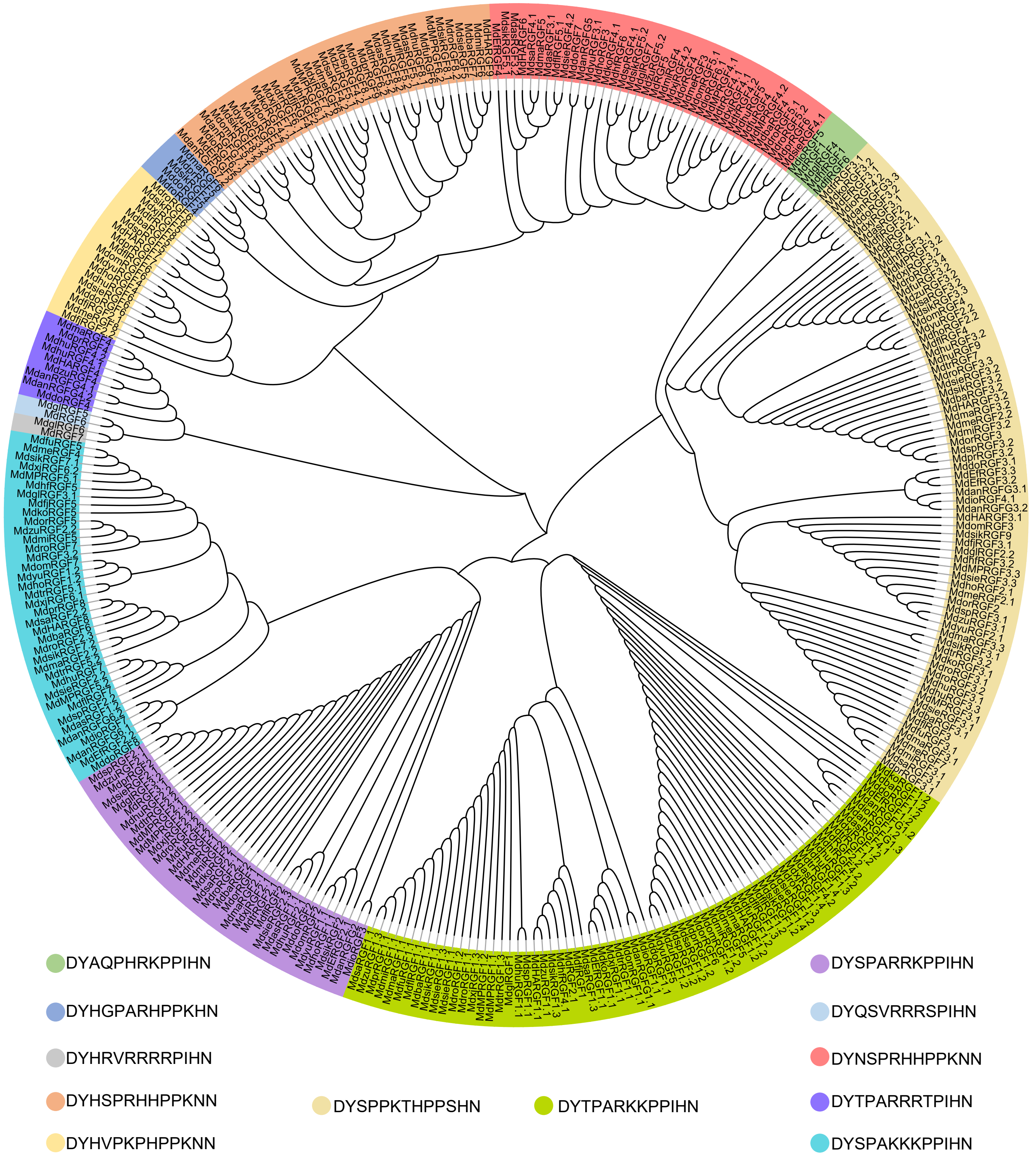

Figure S5. Evolution of the RGF Peptide Family in Malus Genus.The abbreviations of *RGF* family genes correspond to the following Malus species: "Mdfj" denotes *Malus domestica* var. Fuji, "Mdgl" denotes *Malus domestica* var. Gala, "Md" denotes *Malus domestica var.* Golden Delicious, "Mdhf" denotes *Malus domestica* var. Hanfu, "Mdan" denotes *Malus angustifolia*, "Mdhu" denotes *Malus hupehensis*, "Mdtr" denotes *Malus transitoria*, "MdMP" denotes *Malus* × *platycarpa*, "Mdas" denotes *Malus asiatica*, "Mdxj" denotes *Malus baccata* var. xiaojinensis, "Mdro" denotes *Malus rockii*, "Mdsie" denotes *Malus sieboldii*, "Mdsik" denotes *Malus sikkimensis*, "MdEf" denotes *Eriolobus florentinus*, "Mdba" denotes *Malus baccata*, "Mddo" denotes *Malus doumeri*, "Mdfl" denotes *Malus floribunda*, "Mdfu" denotes *Malus fusca*, "MdHA" denotes *Malus halliana*, "Mdho" denotes *Malus honanensis*, "Mdio" denotes *Malus ioensis*, "Mdko" denotes *Malus komarovii*, "Mdma" denotes *Malus mandshurica*, "Mdme" denotes *Malus melliana*, "Mdmi" denotes *Malus micromalus*, "Mdom" denotes *Malus ombrophila*, "Mdor" denotes *Malus orientalis*, "Mdpr" denotes *Malus prunifolia*, "Mdsa" denotes *Malus sargentii*, "Mdsp" denotes *Malus spectabilis*, "Mdyu" denotes *Malus yunnanensis*, and "Mdzu" denotes *Malus zumi*.

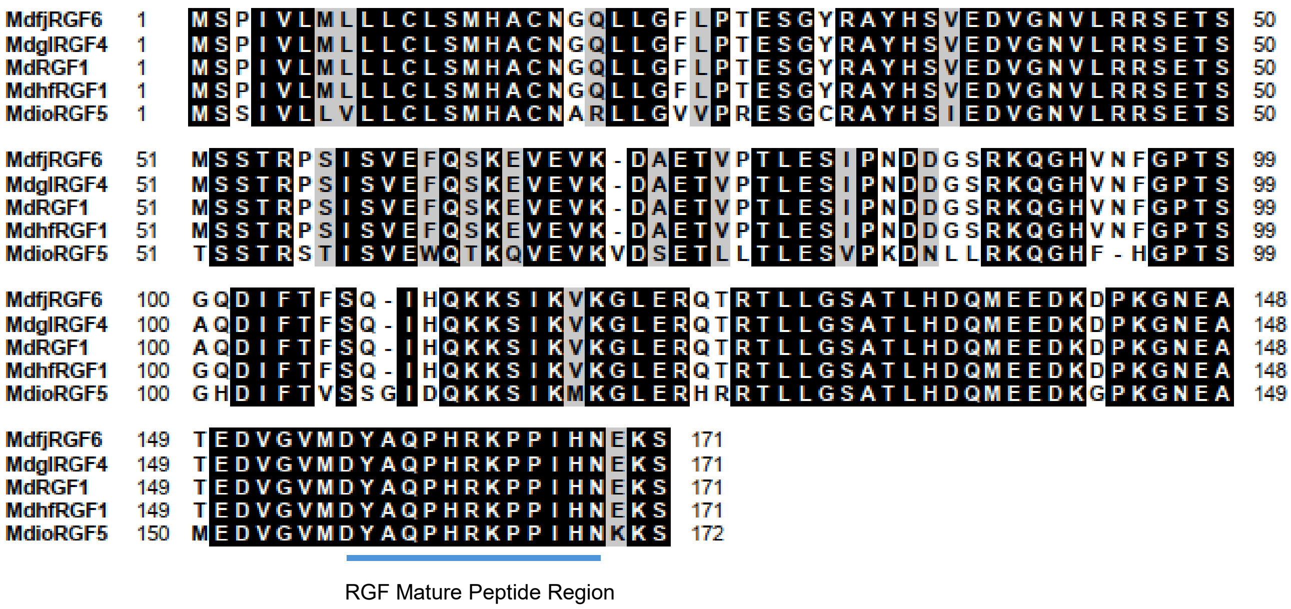

Figure S6. Amino acid sequence alignment of RGF precursor peptides from *Malus* accessions.This panel displays the amino acid sequence alignment of RGF precursor peptides derived from cultivated *Malus* varieties and *Malus* ioensis; the RGF Mature Peptide Region is indicated beneath the alignment.

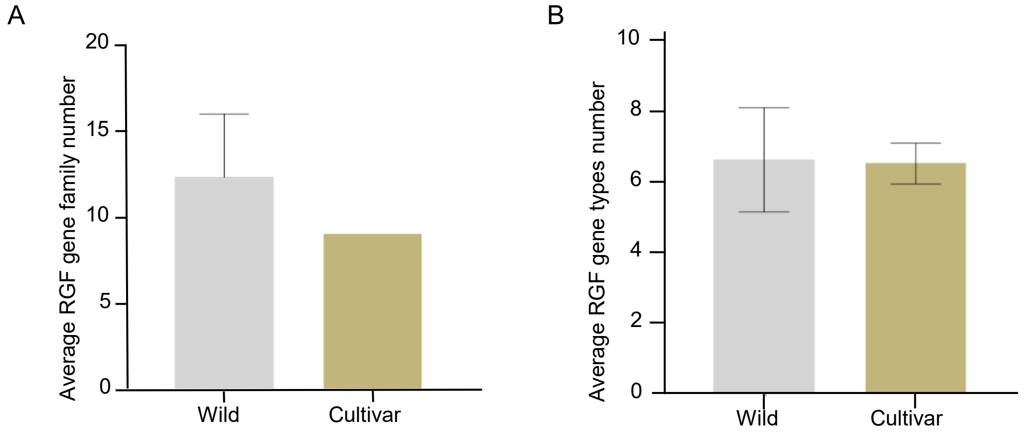

Figure S7. Quantification of *RGF* gene family characteristics across *Malus* groups.(A) Bar plot showing the average *RGF* gene family number in wild and cultivated *Malus* accessions (B) Bar plot showing the average *RGF* gene type number in wild and cultivated *Malus* accessions

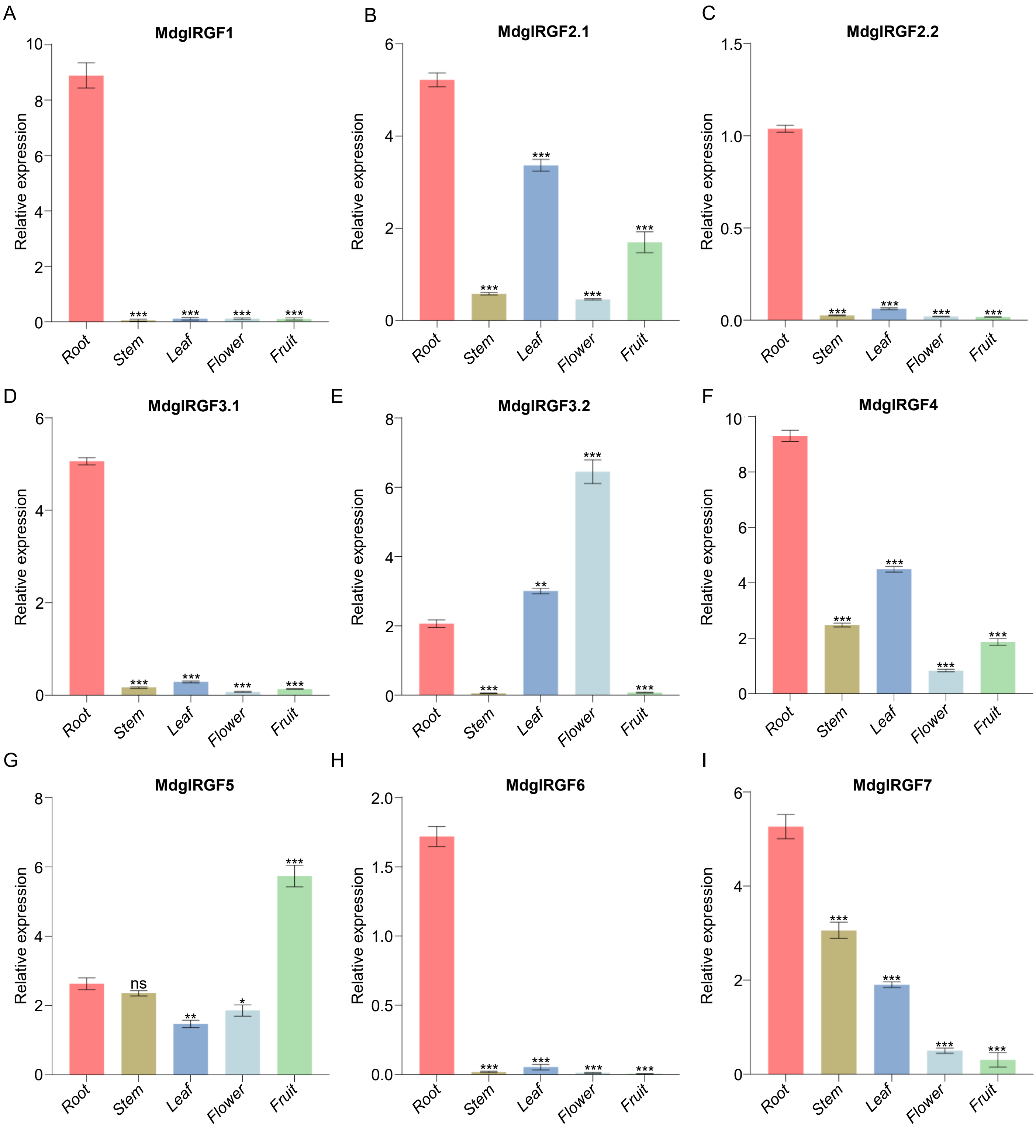

Figure S8. Tissue-specific expression profiles of MdglRGF family genes in *Malus domestica var. Gala.*Relative expression levels of MdglRGF1. (A) MdglRGF2.1 (B) MdglRGF2.2 (C) MdglRGF3.1 (D) MdglRGF3.2 (E) MdglRGF4 (F) MdglRGF5 (G) MdglRGF6 (H) MdglRGF7 (I) in root, stem, leaf, flower, and fruit tissues were determined by quantitative real-time PCR (qRT-PCR). Expression levels were normalized to the reference gene MdActin . Data are presented as mean ± SD (n = 3 biological replicates). Statistical significance was assessed by one-way ANOVA followed by Duncan's post-hoc test: P < 0.05, *P < 0.01, **P < 0.001; "ns" indicates no significant difference.

A

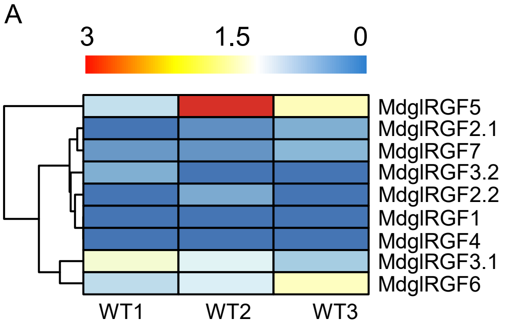

B

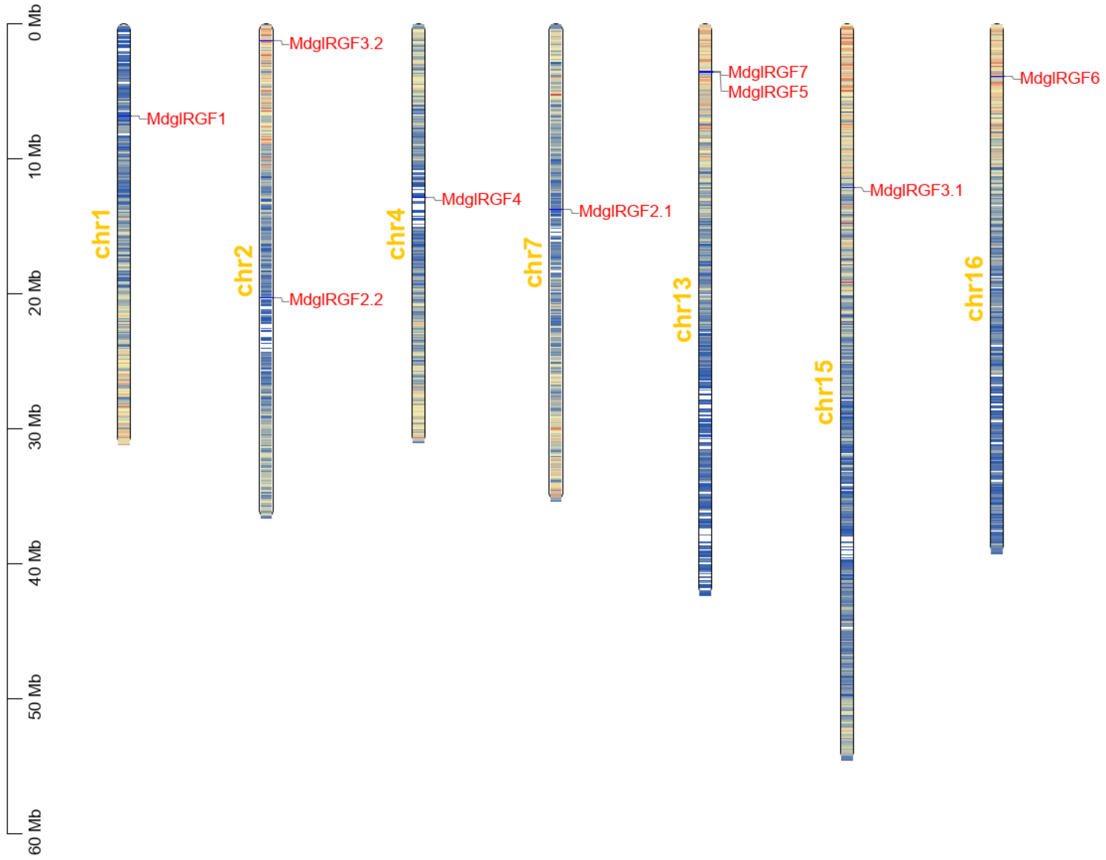

Figure S9. Expression profiles and chromosomal localization of *MdgIRGF* genes in *Malus domestica* ‘Gala’.(A) Expression profiles of *MdgIRGFs* in different tissues. Heatmap shows the relative expression levels (color gradient: red = high, blue = low) in three tissues: WT1 (root), WT2 (stem), and WT3 (leaf). The left dendrogram indicates the clustering of *MdgIRGFs* based on expression patterns. (B) Chromosomal localization of *MdgIRGF* genes in the ‘Gala’ genome. This panel displays the distribution of *MdgIRGF* genes across chromosomes (chr1, chr2, chr4, chr7, chr13, chr15, chr18) of the *Malus domestica* ‘Gala’ genome, with *MdgIRGF* gene names labeled at their corresponding chromosomal positions.

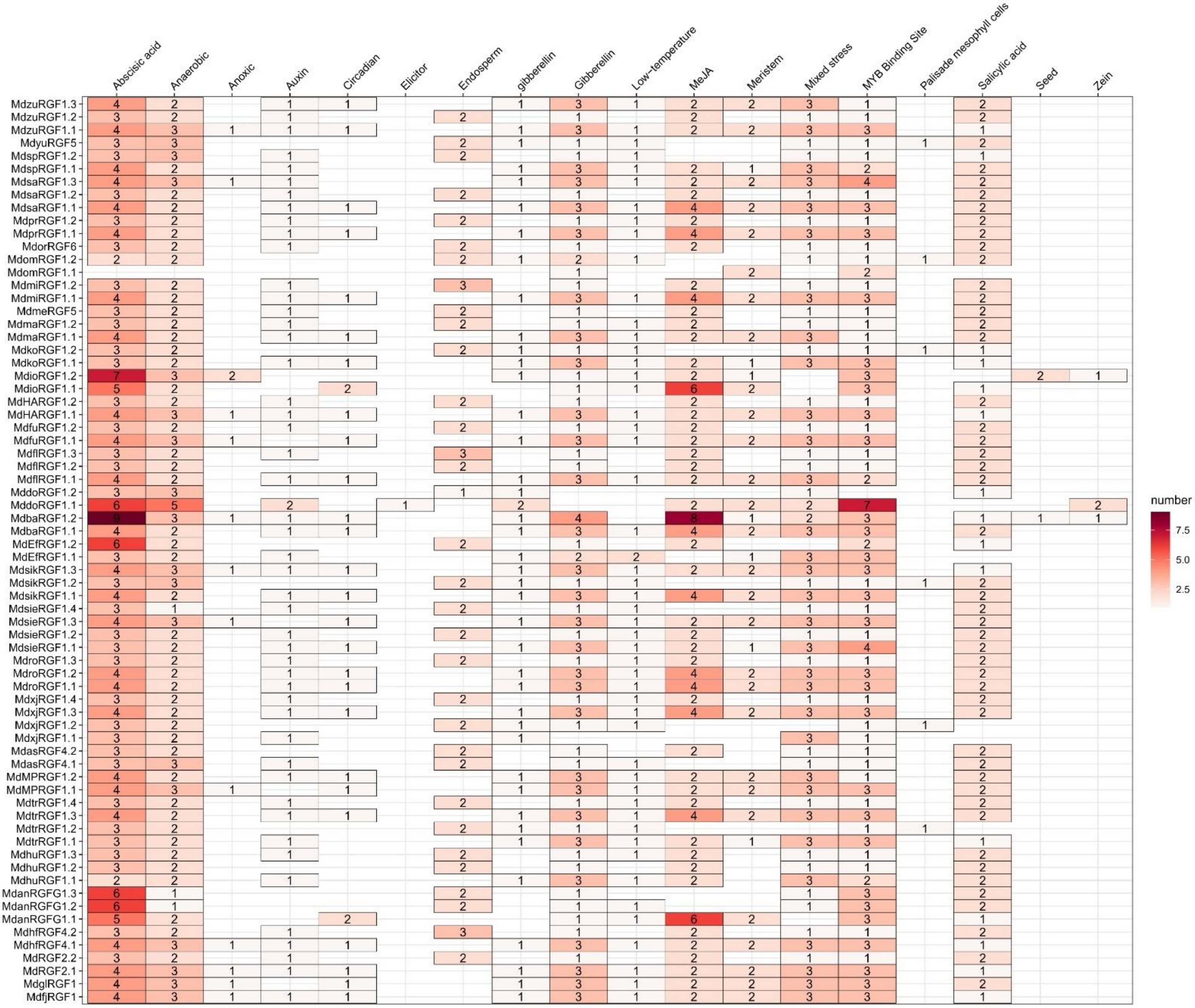

Figure S10. Distribution of cis-regulatory elements in the promoter regions of *Malus RGF* genes.Heatmap showing the count of cis-regulatory elements in the promoter regions of individual *Malus RGF* genes; the elements correspond to signals including Abscisic acid, Anaerobic, Anoxic, Auxin, Cell cycle, Circadian, Endosperm, Gibberellin, Low temperature, MeJA, Meristem, Mixed stress, and MYB Binding Sites. The color gradient (as indicated) represents the number of each cis-regulatory element.

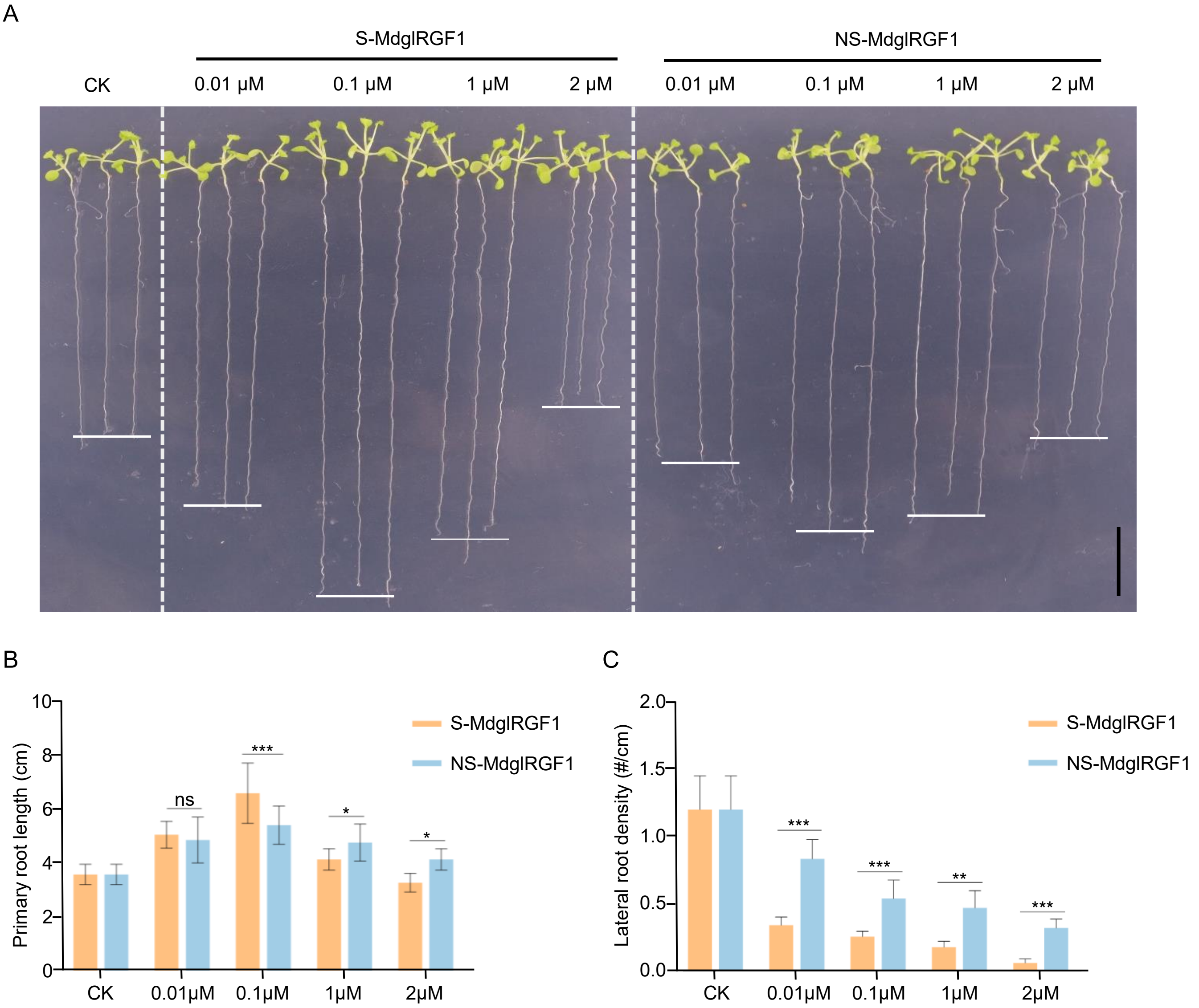

Figure S11. Effects of MdgIRGF1 peptide treatments on *Arabidopsis thaliana* root development.(A) Phenotypic images of *A. thaliana* seedlings under treatments with S-MdgIRGF1 and NS-MdgIRGF1 peptides (concentration gradient: 0.01 μM, 0.1 μM, 1 μM, 2 μM) and control (CK)(Bar = 1 cm). (B) Bar plot showing the primary root length of *A. thaliana* seedlings across S-MdgIRGF1 and NS-MdgIRGF1 treatment groups (including control and concentration gradients); "ns" indicates non-significant difference. (C) Bar plot displaying the lateral root density of *A. thaliana* seedlings across S-MdgIRGF1 and NS-MdgIRGF1 treatment groups (including control and concentration gradients).

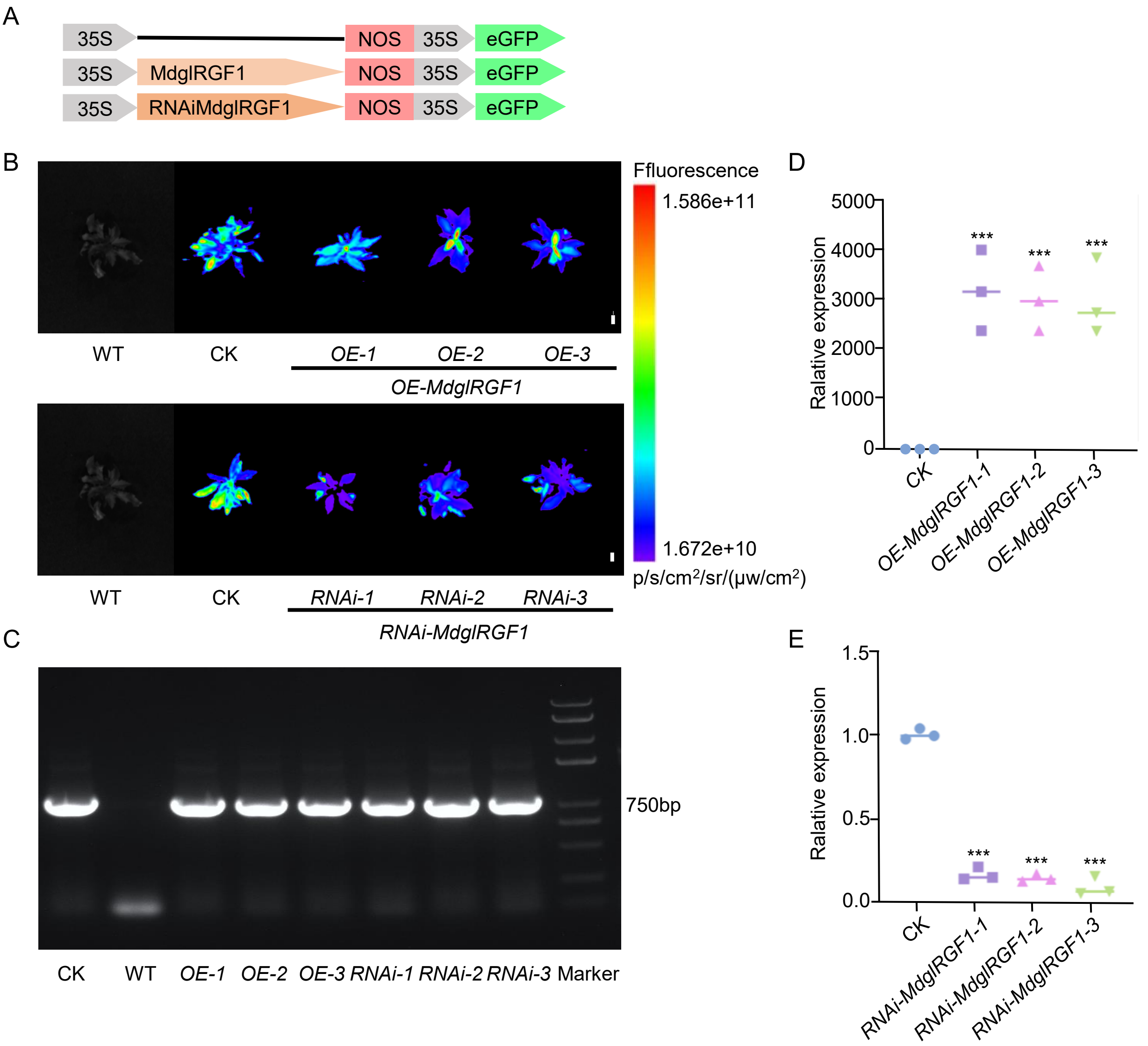

Figure S12. Generation and characterization of *MdgIRGF1*-overexpressing and RNA interference transgenic 'Gala' plants

(A) Schematic of the T-DNA regions of binary vectors used for Agrobacterium-mediated transformation. All constructs contain the CaMV 35S promoter (35S), NOS terminator, and an eGFP reporter gene. They harbor an empty cassette (CK, control), the *MdgIRGF1* coding sequence (*OE-MdgIRGF1*), or an *MdgIRGF1*-specific RNA interference cassette (*RNAi-MdgIRGF1*). (B) Detection of eGFP fluorescence in 3-week-old transgenic plantlets. Fluorescence images of wild-type (WT), CK, three independent *OE-MdgIRGF1* lines (1–3), and three independent *RNAi-MdgIRGF1* lines (1–3) are shown. Scale bar = 1 cm. The color scale indicates fluorescence intensity (units: p/s/cm^2/sr/(μw/cm^2)). (C) PCR verification of transgene integration. Genomic DNA from the indicated lines was amplified using eGFP-specific primers. The expected amplicon confirms the presence of the T-DNA. (D) Relative expression levels of *MdgIRGF1* in *OE-MdgIRGF1* lines. Transcript levels were determined by qRT-PCR in three independent lines with three biological replicates each. Data are normalized to the expression level in the CK line (set to 1). Error bars represent SD. (E) Relative expression levels of *MdgIRGF1* in *RNAi-MdgIRGF1* lines. Transcript levels were determined by qRT-PCR in three independent lines with three biological replicates each. Data are normalized to the expression level in the CK line (set to 1). Error bars represent SD.For (D) and (E), ***P < 0.001 (one-way ANOVA with Tukey's post-hoc test).

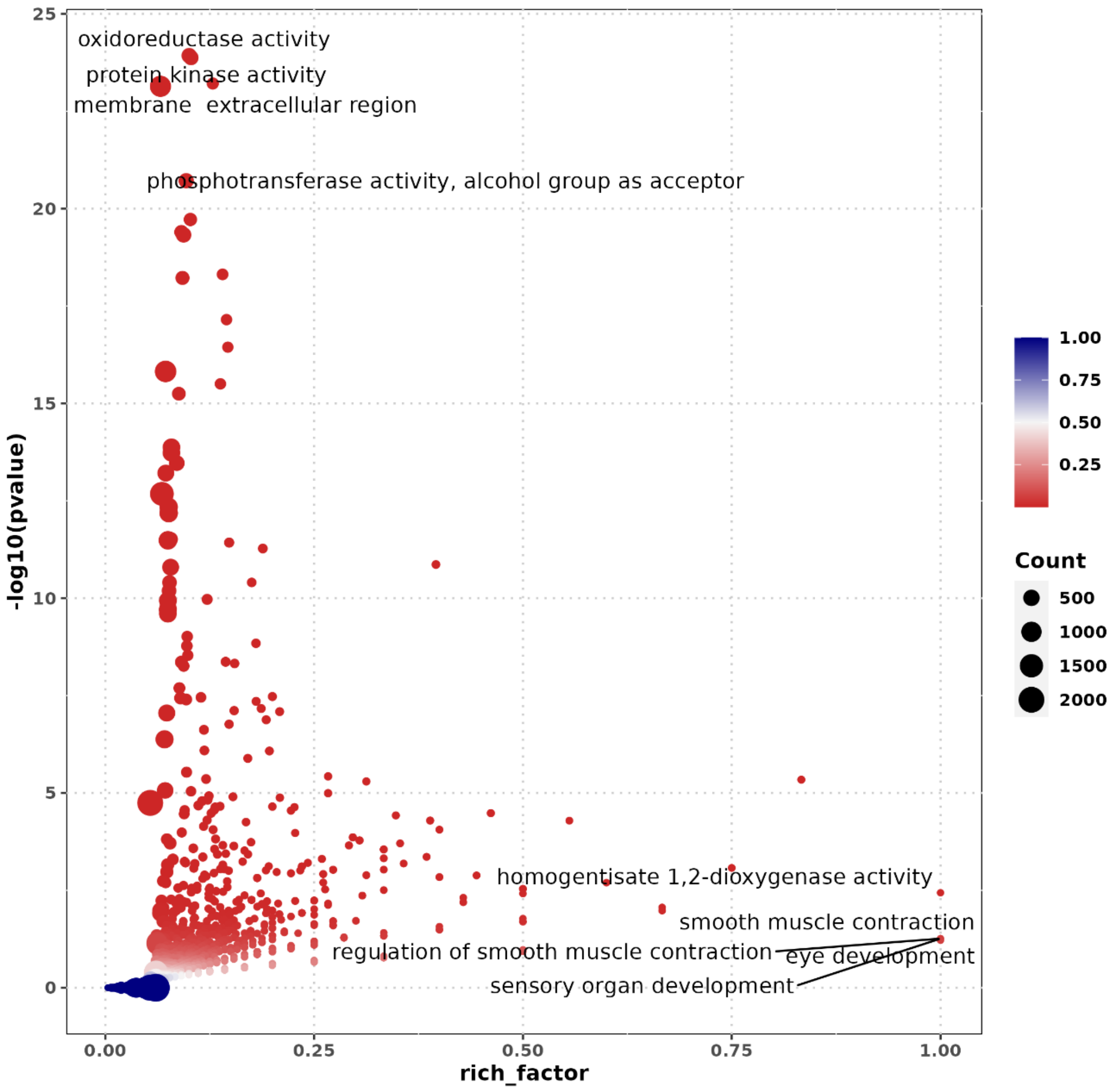

Figure S13. GO enrichment bubble plot of differentially expressed genes in *MdglRGF1*-overexpressing 180-day-old 'Gala' roots.(A) Bubble plot displaying Gene Ontology (GO) enrichment results for differentially expressed genes from *MdglRGF1*-overexpressing 180-day-old 'Gala' roots. The x-axis corresponds to the rich factor, and the y-axis represents -log10 (pvalue). Bubble color (ranging from 0.25 to 1.00, as indicated) and size (matching gene count: 500 to 2000, as indicated) denote the respective parameters; selected GO terms are labeled on the plot.

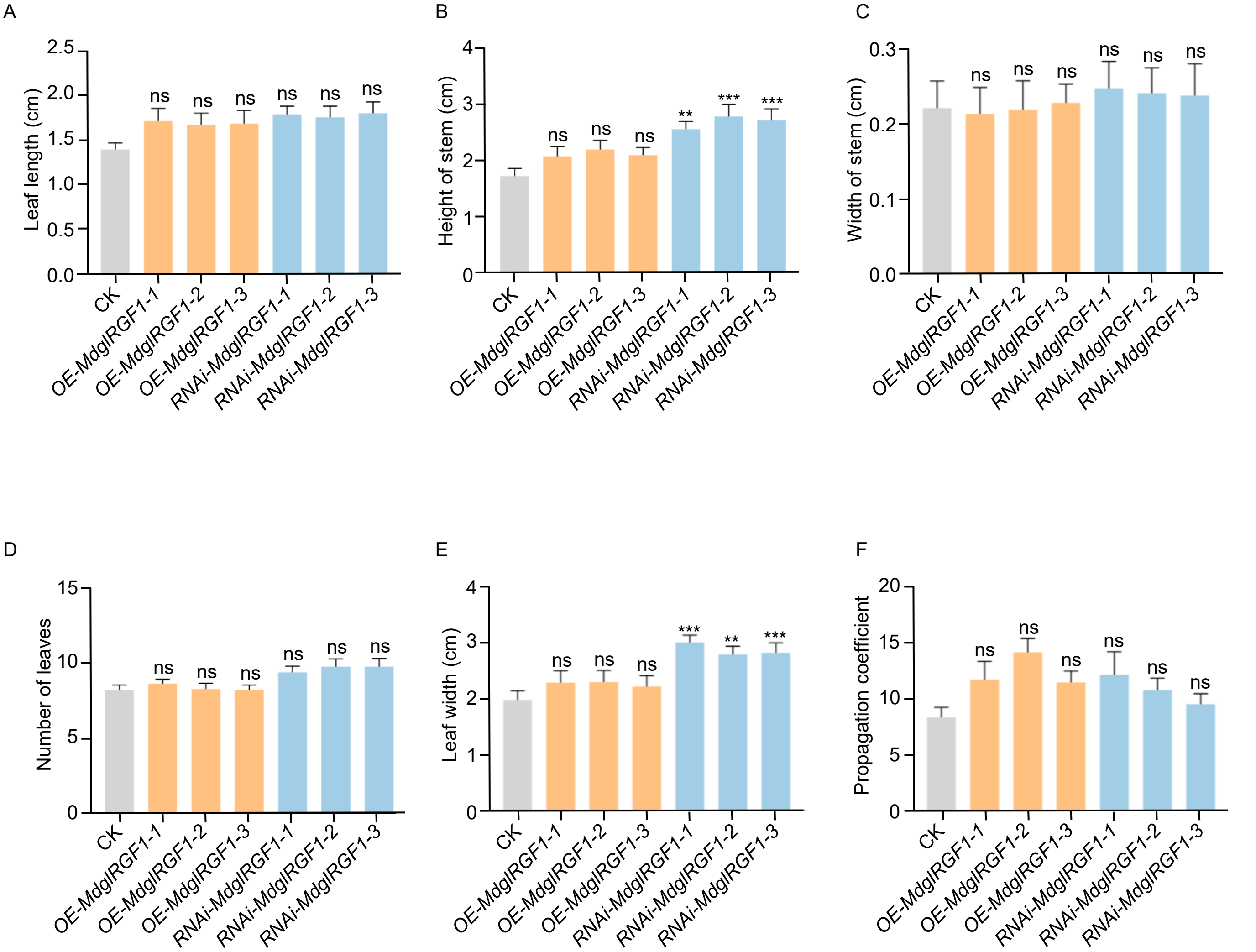

Figure S14. Phenotypic parameter measurements of 30-day-old *MdglRGF1*-overexpressing transgenic plantlets under rootless culture conditions

This figure presents measurements of phenotypic traits in *MdglRGF1*-overexpressing transgenic plantlets (cultured without roots for 30 days), including canopy diameter, plant height, leaf length-to-width ratio, stem height, stem width, leaf width, leaf number, and propagation coefficient. (A) Bar plot showing canopy diameter of the transgenic plantlets. (B) Bar plot displaying plant height of the transgenic plantlets. (C) Bar plot of leaf length-to-width ratio in the transgenic plantlets. (D) Bar plot presenting stem height of the transgenic plantlets. (E) Bar plot of stem width in the transgenic plantlets. (F) Bar plot showing leaf number of the transgenic plantlets.

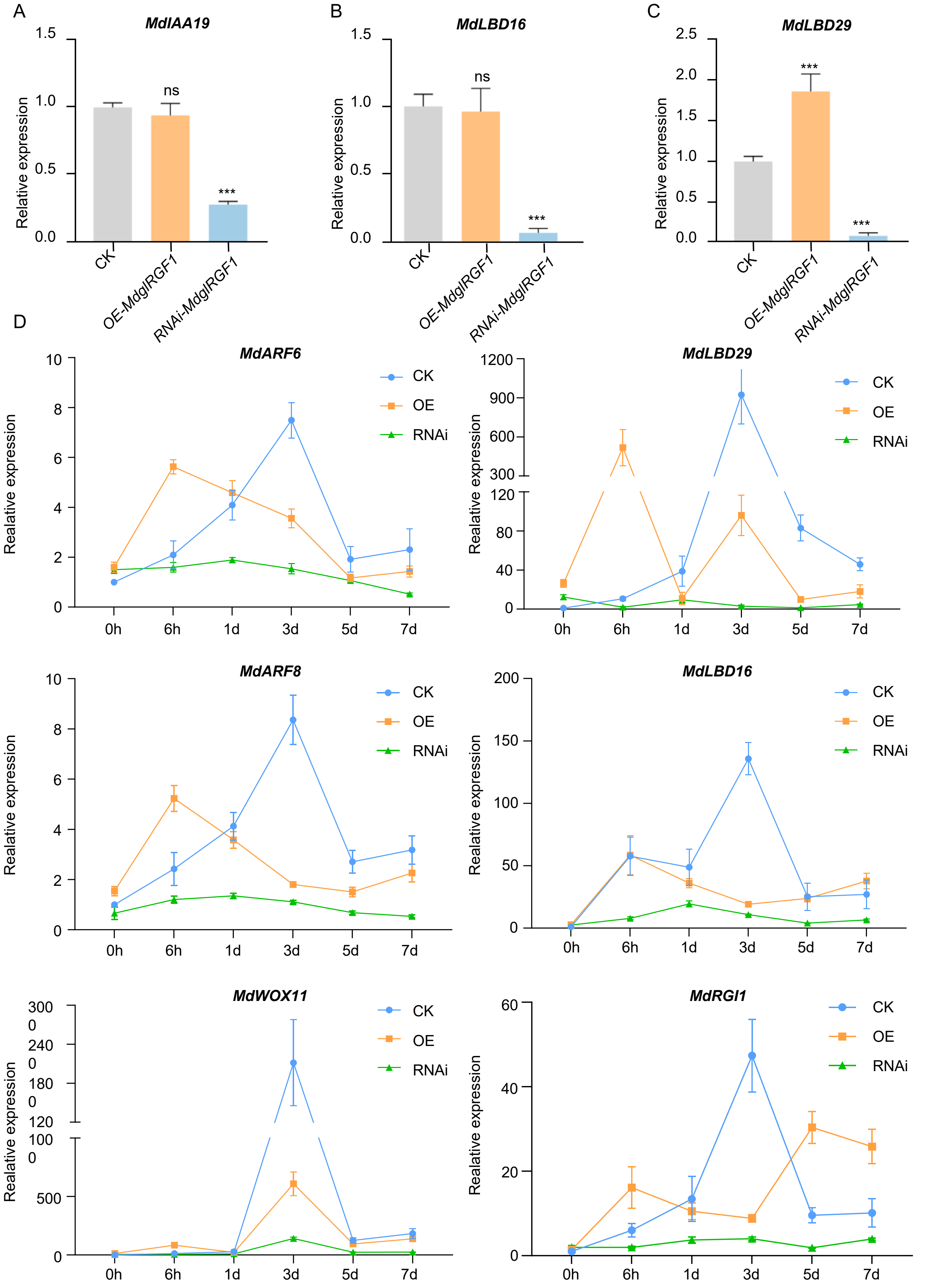

Figure S15. (A–C) RT-qPCR analysis of auxin pathway-related genes in adventitious roots of 30-day-old *MdglRGF1* transgenic apple plants. (A) Relative expression levels of the auxin marker genes *MdIAA19*, *MdLBD16*, and *MdLBD29*. (D) RT-qPCR analysis of adventitious root–related genes in tissues located within 1 cm on both sides of the cutting wound at different time points after cutting. The treatments included empty vector control (CK), *MdglRGF1* overexpression line (*OE-MdglRGF1*), and *MdglRGF1* RNA interference line (*RNAi-MdglRGF1*). significance denoted as $P < 0.05$ (*), $P < 0.01$ (**), $P < 0.001$ (***), and non-significant (ns).

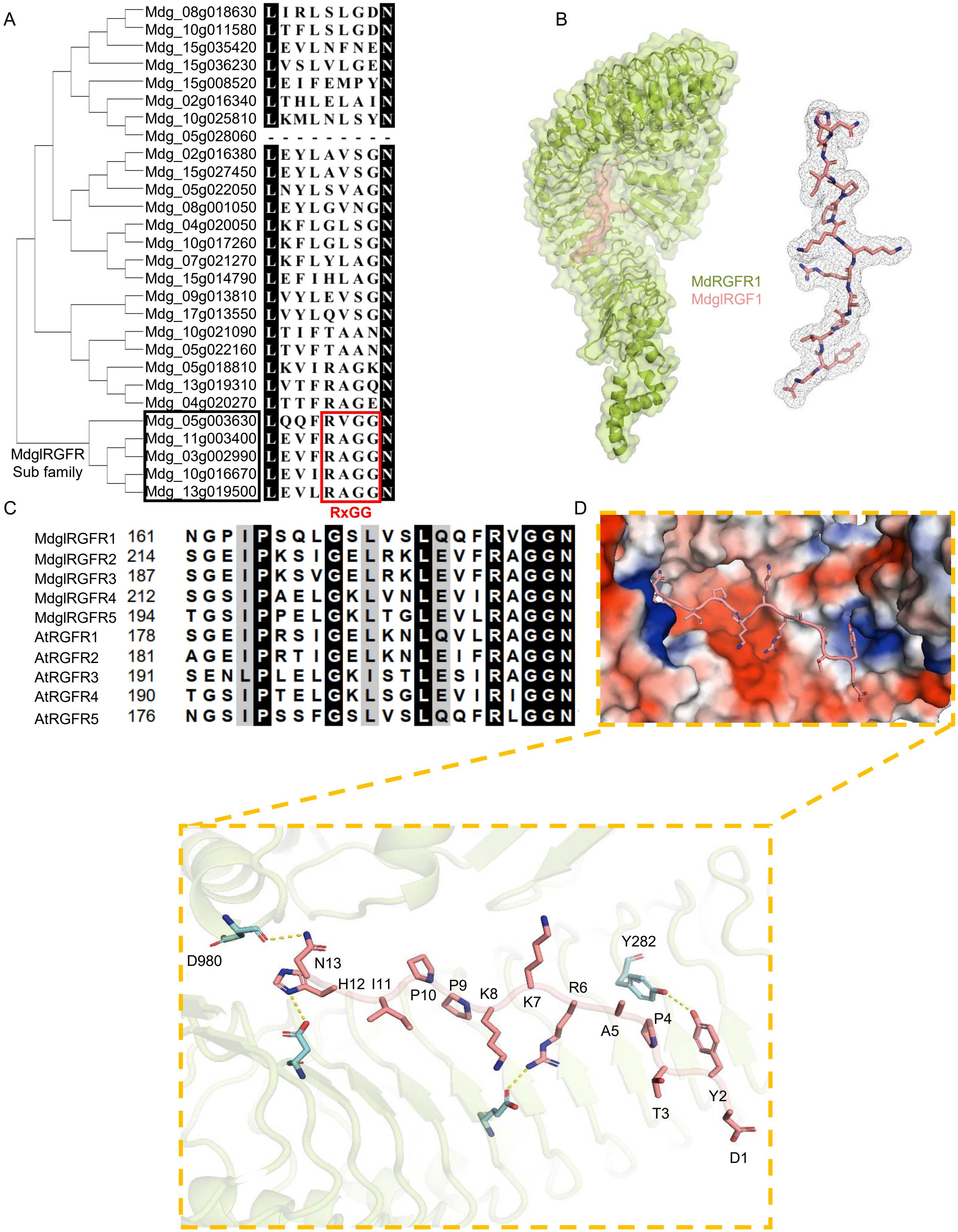

Figure S16. (A)Recognition mechanism of MdgIRGF1 by MdgIRGFR1. Sequence alignment of the LRRs containing the RxGG motif of LRR-RKs in Malus domestica var. 'Gala'. The RxGG motif is highlighted within the red square. Five genes containing the RxGG motif were identified as receptors of MdgIRGFs and named MdgIRGFRs 1-5, and the subfamily of these five genes was named MdgIRGFR subfamily. (b) The binding mode of the RGFR1-RGF complex, with the polypeptide shown in pink and the RGFR1 protein in green in the figure.(C) Amino Acid Sequence Alignment of RGF Receptors in Malus;(D) Multiple key residues of RGF peptides are involved in binding wih RGFR1, and the yellow dashed lines indicate hydrogen bonds.
